# Mapping endometrial vascular functional gradients using depth-derived ultrasound localization microscopy (D-ULM) for early stratification of postinjury fibrotic remodeling and therapeutic guidance

**DOI:** 10.7150/thno.138622

**Published:** 2026-08-12

**Authors:** Xiaowen Liang, Le Gao, Zhili Guo, Meng Du, Yue Pan, Yixiang Lian, Yu Qiang, Haijun Luo, Ying Zhang, Xiaoyan Kui, Hairong Zheng, Weibao Qiu, Zhiyi Chen

**Affiliations:** 1 School of Computer Science and Engineering, Central South University, Changsha, 410083, China.; 2 Key Laboratory of Medical Imaging Precision Theranostics and Radiation Protection, College of Hunan Province, the Affiliated Changsha Central Hospital, Hengyang Medical School, University of South China, Changsha, 410004, China.; 3 Institute of Medical Imaging, Hengyang Medical School, University of South China, Hengyang, 421001, China.; 4 University of Chinese Academy of Sciences, Beijing, 101408, China.; 5 Shenzhen key laboratory of ultrasound imaging and therapy, State Key Laboratory of Biomedical Imaging Science and System, Shenzhen Institutes of Advanced Technology, Chinese Academy of Sciences, Shenzhen, 518055, China.; 6 Institute for Future Sciences, University of South China, Changsha, 410008, China.; 7 Department of Pathology, the Affiliated Changsha Central Hospital, Hengyang Medical School, University of South China, Changsha, 410004, China.

**Keywords:** ultrasound localization microscopy, depth-derived feature, endometrial injury, hierarchical clustering, microvasculature

## Abstract

**Rationale:**

Endometrial injury exhibits significant clinical heterogeneity in fibrotic outcomes. Recovery of microvascular perfusion is central to prognosis, while the depth of injury is an important factor influencing regenerative capacity. However, current clinical imaging techniques are limited by diffraction, resulting in insufficient resolution to reliably depict depth-dependent microvascular architecture of the endometrium, with consequent difficulty in evaluating injury depth or in making early prognostic distinctions.

**Methods:**

A depth-derived ultrasound localization microscopy (D-ULM) approach is introduced to capture depth-dependent microvascular features in the endometrium. First, we established a normal depth reference band from healthy rats to provide a physiological baseline for identifying pathological vascular changes. Next, in an endometrial injury rat model, we applied unsupervised clustering of early D-ULM features to identify microcirculation phenotypes. Subsequently, we evaluated the correlation between D-ULM features, D-ULM clusters, and late-stage fibrosis area as well as immunofluorescence markers of hypoxia and inflammation, with a view to tying early microvascular changes to eventual recovery or to fibrotic remodeling.

**Results:**

In healthy rats, depth-profile curves revealed a distinct transition zone between the deep and superficial layers, enabling the delineation of a quantifiable functional boundary of the endometrial microvasculature. Based on this depth-dependent layering, normal reference intervals were established for the seven D-ULM features. Among these, the reference interval for the vascular distribution center (com_depth_vessel) was 0.371–0.481. In injured cohort, unsupervised hierarchical clustering of day-3 D-ULM features identified three phenotypes: regenerative (Reg), inflammatory hyperperfusion (IH), and irreversible destruction (ID). The day-14 fibrosis area differed significantly among the phenotypes (*P* < 0.001), with the Reg phenotype showing the lowest fibrosis, while the IH and ID phenotypes exhibited markedly higher levels. Several D-ULM features were positively correlated with day-14 fibrosis, including com_depth_vessel (ρ = 0.707, q < 0.001), vessel_slope (ρ = 0.539, q = 0.017), vessel_auc (ρ = 0.811, q < 0.001), and mean_vessel_shallow (ρ = 0.846, q < 0.001); conversely, large_ratio_diff was inversely correlated (ρ = -0.568, q = 0.011). The IH phenotype exhibited elevated M1/M2 ratio, HIF-1α, and CD31 expression, whereas the ID phenotype showed increased M1/M2 ratio and HIF-1α but reduced CD31 expression by immunofluorescence.

**Conclusions:**

D-ULM enables depth-resolved imaging of endometrial microvascular functional gradients in a noninvasive manner. This approach provides an imaging framework for early stratification of injury phenotypes, and may guide personalized preventive strategies for patients at risk of postinjury fibrosis.

## Introduction

Endometrial fibrosis, one of the leading causes of female infertility, arises secondary to uterine injury caused by intrauterine infections, recurrent miscarriages, or surgical procedures, affecting nearly 50% of patients who undergo procedures such as induced abortion or hysteroscopic surgery 1,2. In its mid-to-late stage, this condition can progress to intrauterine adhesions (IUAs), leading to amenorrhea and loss of fertility, thus representing a major burden on female reproductive health. Clinically, one major challenge is the marked individual variation in the progression of fibrosis 3. It is crucial to elucidate the underlying mechanisms and to develop precise detection methods for identifying individuals at high risk of fibrosis, so as to enable early assessment, timely intervention, and improved prognosis.

While the precise mechanism of endometrial fibrosis has not been fully elucidated, numerous findings support the view that the depth of endometrial injury, along with structural and functional changes of the microvasculature, is closely associated with both regenerative ability and post-injury fibrosis of the endometrium 4. Superficial injury without involvement of the spiral arteriole-related stem or progenitor cell niche in the basal layer appears to favor functional regeneration, but deep injury, such as loss of the vascular reservoir or damage to the subepithelial capillary plexus, shifts the balance toward pathological repair and subsequent fibrosis 5,6. Early assessment of **f**ull-thickness endometrial microvessels is valuable for predicting outcome and guiding treatment decisions, yet current clinical imaging modalities face limitations in visualizing the superficial endometrial microvasculature. MRI gives only submillimeter to millimeter resolution. Color Doppler is not trustworthy for vessels under about 0.1 mm, nor is it sensitive to very slow flow 7. Microvascular imaging or superb microvascular imaging helps with detection of small vessels by eliminating artifacts due to motion and to rapid flow, but they do not escape the basic diffraction limitation 8. Regarding contrast-enhanced ultrasound, microbubbles contribute to a higher sensitivity of detection yet do not allow one to escape the same physical resolution limit 9. Photoacoustic imaging is of high resolution but is limited in terms of penetration depth 10. The photoacoustic endoscope may overcome this penetration limitation by imaging from within the uterine cavity, but it still requires intrauterine probe placement, which risks additional injury or infection of the endometrium 11.

Ultrasound localization microscopy (ULM) is a recently developed noninvasive method of super-resolution ultrasound. By tracking intravascular microbubbles, ULM goes beyond the diffraction limit and yields micron-sized (approximately 10 μm) resolution, thus enabling quantitative assessment of microcirculation 12,13. It has proven of great value for deep-tissue imaging and has already been applied to microvascular analysis of various organs (brain 14, heart 15, and kidney 16). With its high resolution and quantitative parameters, ULM can precisely capture alterations in the full-thickness endometrial microperfusion network and help reveal the intrinsic relationship between early microcirculatory dysfunction and endometrial fibrogenesis. Notably, traditional ULM analysis has primarily focused on average parameters within regions of interest (ROIs), capturing microperfusion differences (e.g., density, velocity, tortuosity) between normal and pathological areas 17. However, the assessment and prognosis of endometrial injury are highly related to the depth of injury. A global averaging approach based on ROIs is likely to obscure critical variations along the depth axis, particularly the gradient of vascular parameters from the myometrial side to the luminal side, a gradient that inherently carries the functional zonation information of the endometrium.

**Figure 1:**
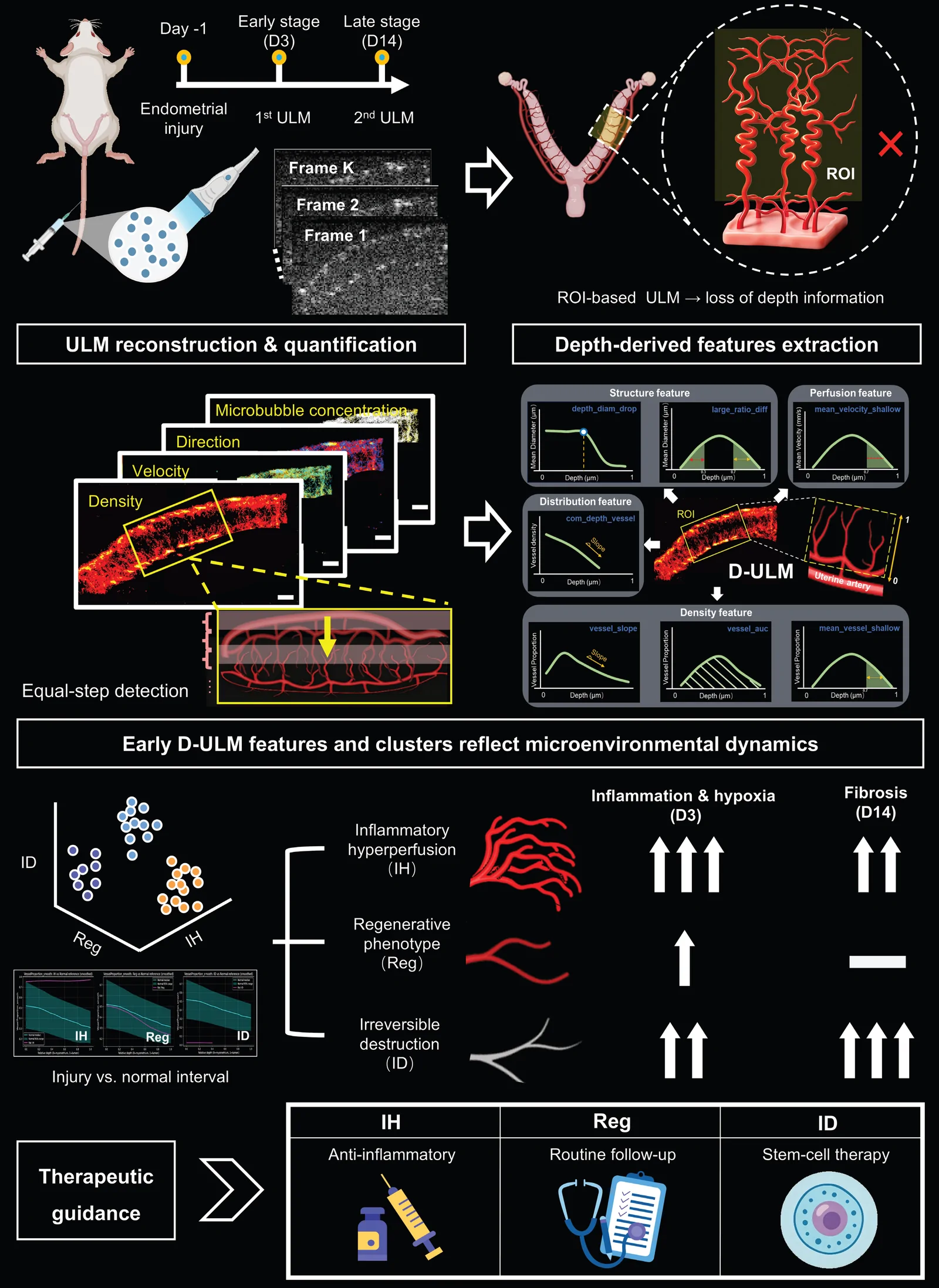
** Schematic overview of the D-ULM workflow.** ULM imaging was conducted on day 3 (D3) and day 14 (D14) following endometrial injury. Equal-step sampling was applied to extract depth-derived ULM features, thus yielding seven D-ULM features. Normal reference intervals were determined in healthy rats. Among injured rats, hierarchical clustering of D3 D-ULM features identified three phenotypes: a regenerative phenotype, an inflammatory hyperperfusion phenotype, and an irreversible destructive phenotype. Each of these phenotypes was subsequently evaluated for inflammation, hypoxia, angiogenesis, and fibrosis severity (using pathological imaging). Distinct D3 D-ULM phenotypes reflect changes in the endometrial microenvironment, potentially offering early guidance for intervention strategies to prevent postinjury fibrosis. Scale bar: 1 mm.

In this study, we report the noninvasive, high-resolution ULM visualization of endometrial microperfusion in healthy and injured rats. Importantly, recognizing both the physiological importance of depth-dependent vascular heterogeneity and its pathophysiological relevance to endometrial injury, we innovatively develop a depth-derived ULM (D-ULM) framework (**Figure 1**). The present framework is designed to extract vertical features that reflect changes in microvascular parameters with respect to endometrial depth, and to clarify the basic connection of early microperfusion disturbances with later fibrosis in the injured endometrium. For purposes of physiology-based comparison, a normal depth reference band for D-ULM features was established initially using healthy rats. In the injured cohort, deviations of individual features from the reference band were assessed at the early stage of injury, followed by unsupervised clustering to identify microperfusion phenotypes. These D-ULM features and phenotypes were subsequently validated for their correlation with late-stage fibrotic outcomes and the local immune microenvironment. This work is the first to apply depth-derived ULM analysis for the early stratification of fibrosis risk after endometrial injury. It provides a high-resolution quantitative imaging basis for personalized management of endometrial injury before irreversible fibrosis develops.

## Methods

The study was conducted in three phases. In Phase 1 (normal reference cohort), 30 healthy rats underwent ULM imaging to establish the normal depth reference band. In Phase 2 (injury cohort), an additional 30 rats with random injury severity were included. ULM imaging was performed on day 3 postinjury for depth-derived feature extraction and unsupervised clustering. In Phase 3 (validation), the injured rats were followed up to day 14 for fibrosis outcome assessment (Masson staining) and immune mechanism analysis (immunofluorescence, IF).

### Animal model

In this study, 60 female Sprague-Dawley rats (Hunan SJA Laboratory Animal Technology; 8–10 weeks old; 220–250 g) were used to establish D-ULM normal reference intervals and the endometrial injury model. All rats were housed in a specific-pathogen-free environment with free access to food and water, stable ambient temperature and humidity (23–25 ºC, 50 ± 5%), and a 12-h light/dark cycle. For the injury group, rats in the oestrus phase (confirmed by vaginal cytology using Wright-Giemsa staining) underwent ethanol-induced endometrial injury by intrauterine instillation of 95% ethanol. To simulate clinically variable injury severity, ethanol was retained in the uterine cavity for 30 s, 60 s, or 90 s (n = 10 per group). After modeling, the uterine cavity was thoroughly lavaged with saline. Absorbable sutures were used to close the surgical incisions, after which postoperative analgesia was administered along with a prophylactic dose of antibiotics. All procedures involving animals were reviewed and approved by the Animal Ethics Committee of Hunan Prevention and Treatment Institute for Occupational Diseases (Approval No. HNZFY-2025-011) and were conducted according to the ARRIVE guidelines.

### Ultrasound imaging protocol and data acquisition

The rats were initially anesthetized with 3% isoflurane in an induction chamber and then moved onto a heated imaging platform, on which anesthesia was kept at 1.5% isoflurane during the whole imaging session. A midline abdominal incision was made with the aim of exposing the uterine horns, ensuring accurate probe positioning. B-mode and CDFI were obtained initially by means of ultra-high frequency ultrasound (SonoRover UR820, Hyus Meditec, Shenzhen, China). Power Doppler and ULM imaging were performed on a Vantage 256 system (Verasonics Co., Ltd., USA) and a programmable system (FlexEcho, Hyus Meditec, Shenzhen, China). A high-frequency linear array transducer (15 MHz, 128 elements, Hyus Meditec, Shenzhen, China) was placed on a stereotaxic arm with a holder, positioned above the uterus, and gently coupled to the tissue surface using ultrasound gel. With the uterus fixed and the mid-longitudinal plane determined, microbubbles (Sonovue®, Bracco, Italy) were diluted to 6 × 10^7 MBs/mL in sterile saline and injected via the tail vein at a constant rate of 25 mL/h using a syringe pump (HK-400I, Hawk Medical Instrument Co., Ltd., Shenzhen, China). Imaging acquisition was begun at the time when microbubble concentration had stabilized after the bolus peak. ULM data were acquired using a 5-angle wide-beam compounding sequence, with steering angles ranging from -10° to 10° in 5° increments. The center frequency of the detection pulse was 15 MHz, and the pulse repetition frequency was 1 kHz. A 2-cycle pulse was transmitted with a 100% duty cycle. For each uterine imaging plane, radio frequency (RF) data corresponding to B-mode imaging were acquired in 40 groups, with 400 frames collected per group, resulting in a total of 16,000 frames per imaging plane. For each rat, ULM datasets were acquired from the uterine imaging planes and stored for subsequent offline processing.

### Offline ULM processing

The ULM data processing procedure consisted of several key steps, including singular value decomposition (SVD) filtering, motion correction, microbubble localization, microbubble tracking, and vascular image reconstruction. The acquired RF data were first beamformed using a delay-and-sum approach and then demodulated into IQ signals. Subsequently, SVD filtering was applied to the IQ data to separate the microbubble signals from tissue signals, with the filter threshold parameters set to [15, 400]. To further suppress residual low-frequency tissue components and high-frequency noise, a temporal bandpass filter was applied along the slow-time dimension, with the cutoff frequency range set to 50–250 Hz. To mitigate endometrial motion caused by respiration and cardiac pulsation during long-duration acquisitions, a VoxelMorph-based deep learning method was employed to estimate the nonrigid deformation fields of the endometrium, followed by motion correction to reduce motion-induced artifacts in ULM reconstruction. For microbubble localization, candidate microbubbles were first identified based on regional intensity maxima in the filtered IQ data, followed by subpixel localization using Gaussian curve fitting or radial symmetry localization. The localized microbubbles were subsequently linked into trajectories using a nearest-neighbor tracking strategy, with a maximum linking distance of 1.5 pixels and a minimum trajectory length of 15 frames. Finally, the accumulated trajectories were projected onto a super-resolved grid to reconstruct vascular density images of the rat endometrium, while local velocity maps were generated by averaging trajectory-derived velocities within each pixel. The direction map and microbubble concentration map were subsequently generated.

### ULM imaging analysis

Ultrasound image analysis was performed using MATLAB software (2020b, MathWorks, USA). The reconstructed ULM images and corresponding velocity maps were further analyzed using custom MATLAB scripts. For comprehensive hemodynamic and morphological assessment, ten conventional ULM features were quantified: fractal dimension (FD), vascular proportion, maximum velocity, mean velocity, tortuosity, mean diameter, large vessels, tiny vessels, junction count and branching index (**Table 1**).

Subsequently, two analysis modes were implemented: a direct ROI analysis mode and a centerline-based curve analysis mode. In the direct ROI mode, several freehand ROIs were manually selected, and vascular quantities were calculated separately for each ROI. Each ULM image was analyzed with ROIs independently drawn by two expert, reliable researchers. This mode was used both for extracting standard ROI-based ULM features and for comparing features between the deep and superficial layers. The quantitative tables, histograms, and related visualizations were exported for statistical analysis. In the centerline-based method, one starts by marking the area under study, and then draws a centerline along the endometrium. The inner boundary was defined as the uterine cavity line, and the outer boundary as the endometrium-myometrium junction. For reproducibility, the ROI dimensions (length × depth) were standardized to ≥ 2 × 1 mm; for injured rats, the ROI was confirmed to cover the lesion area. The centerline was resampled at 1-pixel intervals, and all pixels lying in the ROI were projected onto the nearest centerline position. ULM features were computed using sliding windows centered at equally spaced sampling points along the centerline. The sampling positions were defined as 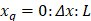, where 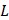
is the total centerline length, and 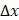
is the sampling interval. The window length was set to 8% of the centerline length and constrained to 0.5–2.5 mm, while 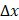
was set to 20% of the window length with a lower bound of 0.2 mm. With these settings, the number of sampling positions was 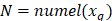, approximately 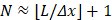, which varied slightly across rats depending on ROI length. At each sampling position, ten conventional ULM features were calculated, generating a centerline-based spatial profile curve for each feature. This mode was subsequently used for extraction of the D-ULM features and for establishment of the normal depth reference band. For an objective analysis of the boundary separating the D-end (deep layer) from the S-end (superficial layer), we applied a multimetric change-point detection framework using piecewise linear regression. After z score normalization, each metric profile was partitioned at candidate positions; the position that minimized the mean residual sum of squares across all metrics was selected as the shared transition boundary. Bootstrap resampling (metric level, 1000 iterations) was performed to estimate the 95% confidence interval of the boundary.

### Assessment of depth-derived vascular features

To quantify the depth-dependent changes in microvascular parameters from the myometrial side to the uterine cavity, we extracted seven features based on the depth-profile curves. Depth normalization was first performed to eliminate interindividual differences in endometrial thickness: the coordinates were linearly transformed to a standardized scale (0 = myometrial side, 1 = uterine cavity side). Let the relative depth 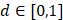, where 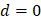
denotes the myometrial side and 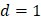
the luminal side. For each rat, the values of a given parameter are measured at equally spaced depth positions 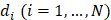. Define:

- Vessel proportion: 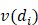
- Mean diameter: 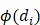
- Large vessel diameter: 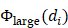, tiny vessel diameter: 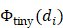
- Mean blood flow velocity: 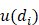
(mm/s)

All depth values are normalized to the [0, 1] interval to account for inter-individual variations in endometrial thickness. The features 1–3, 5, 6 are primarily structural, whereas features 4 and 7 are perfusion-oriented. Numerical calculations were performed using linear interpolation for curve resampling, finite differences for derivative estimation (e.g., for depth_diam_drop), and the trapezoidal rule for area integration (e.g., for vessel_auc). For features that rely on derivative-based identification, if the identified point falls near the boundaries (< 0.1 or > 0.9), we attempt to use the zero-crossing of the second derivative as an alternative; otherwise, the feature value is set to NaN.

#### **1. Center-of-mass depth of vessel proportion** (com_depth_vessel)



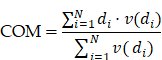



This is the first moment (i.e., the weighted average depth) of the vessel proportion curve, indicating the central location of microvascular distribution along the endometrial depth.

#### **2. Slope of vessel proportion versus depth** (vessel_slope)



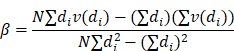



The linear regression slope of vessel proportion on relative depth, reflecting the rate of density decline from the myometrial side toward the uterine cavity side.

#### **3. Area under the vessel proportion curve** (vessel_auc)



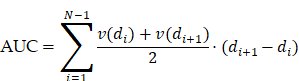



Computed by the trapezoidal rule, this area represents the average vascular density over the normalized depth.

#### **4. Mean vessel proportion in the shallow zone** (mean_vessel_shallow)



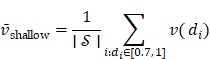



where 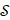
is the set of points with 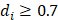. This quantifies the vascular density of the superficial layer (the luminal side 30% of the endometrium).

#### **5. Depth of the steepest drop in mean diameter** (depth_diam_drop)



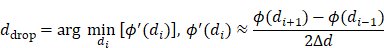



The relative depth corresponding to the minimum of the first derivative of the mean diameter curve, marking the transition from larger arteries to the capillary network.

#### **6. Difference in large-vessel ratio between deep and shallow zones** (large_ratio_diff)

First, the large-vessel ratio at a given depth is defined as:



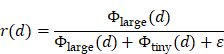



where 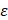
is a small constant to avoid division by zero. Then:



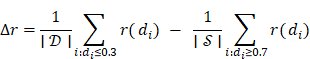



where 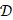
denotes points in the deep zone (0–30% depth) and 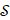
the shallow zone (70–100% depth). This difference captures the depth gradient of a large-vessel proportion.

#### **7. Mean blood flow velocity in the shallow zone** (mean_velocity_shallow)



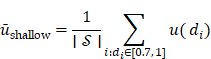



This is the average blood flow velocity within the functional layer (shallow zone), expressed in mm/s, reflecting functional layer perfusion.

### Unsupervised hierarchical clustering analysis

Unsupervised hierarchical clustering (Ward’s linkage, Euclidean distance) was applied to the day-3 D-ULM features of injured rats. The optimal number of clusters was estimated using the silhouette coefficient. For visualization of the clustering pattern, principal component analysis (PCA) was employed to map the high-dimensional data onto the first two principal components. The normalized mean values of each cluster are displayed in a radar chart, and a hierarchical clustering heatmap was used to represent the various patterns of feature expression among all samples. To assess whether early clustering reflected mid-to-long-term blood flow and functional recovery, all rats were followed longitudinally until day 14. For each cluster, typical ULM density and velocity maps were formed. Diameter distribution and velocity distribution curves from day 3 to day 14 were analyzed and compared. Two representative features, including vessel proportion (which reflects vascular density) and branching index (which reflects network complexity), were quantitatively compared across clusters. To assess whether the D-ULM-derived clusters reflect distinct and reproducible patterns, an expert blinded to the clustering assignments independently analyzed the ULM images. The expert-based classification served as a tentative reference for assessing whether the data-driven clusters aligned with the qualitative assessments of vascular recovery patterns. Consistency between the clustering and the expert-based visual categories was assessed using the adjusted Rand index. For the depth profiles of vessel proportion per cluster, the previously defined normal reference band was overlaid on the data to help detect departures from the physiological baseline. The significance of intercluster differences was assessed using Kruskal-Wallis one-way analysis of variance (ANOVA) and Dunn’s post hoc correction. The clusters and their D-ULM features served as candidates for correlation analysis with day-14 fibrosis outcome and for comparison of early-stage immune markers across phenotypes.

### Histopathological validation

With ULM imaging completed, rats were euthanized and the uterine tissues were collected for histology. Tissues were preserved in 4% paraformaldehyde for 24 h, embedded in paraffin, and cut into 5-μm-thick longitudinal slices. Masson trichrome staining performed on day 14 was regarded as the gold standard for measuring fibrosis. For each rat, three non-consecutive sections were taken, and five random fields (400 × magnification) were recorded separately for each section. Regarding collagen area percentage (CAP), ImageJ was used for quantification, and the average value was taken as an index of overall fibrosis severity. As for the histological boundary between the superficial and the deep layers, periodic acid–Schiff (PAS) staining was applied to detect metabolic activity, Ki-67 immunohistochemistry was performed to evaluate proliferation, and H&E staining was used to identify structural differences.

To explore the mechanistic basis of D-ULM-based phenotyping, an additional set of model rats (three per phenotype) was euthanized on day 3 to gather uterine tissues for IF staining, enabling correlation of early D-ULM features with the immune microenvironment. For the IF analysis, parameters such as hypoxia (HIF-1α), angiogenesis (CD31), or inflammation (macrophage types, M1:CD86; M2:CD206) are considered. Nuclei were counterstained with 4’, 6-diamidino-2-phenylindole (DAPI). Images were acquired using a fluorescence microscope (Nikon Eclipse C1, Tokyo, Japan). ImageJ was used to quantify HIF-1α-positive and CD31-positive areas, as well as CD86+ (M1) and CD206+ (M2) macrophage counts for M1/M2 ratio calculation.

### Statistical analysis

All statistical analyses were performed using GraphPad Prism (V10.1.2; GraphPad Software, San Diego, CA, USA) and Python (V3.10.11; Python Software Foundation, Wilmington, DE, USA). Normality of data distribution was assessed using the Shapiro–Wilk test. Normally distributed continuous variables are presented as mean ± standard deviation (SD), whereas non-normally distributed variables are presented as median with IQR. For comparisons between the deep and superficial endometrial layers in normal rats, a paired t-test was used for normally distributed data and the Wilcoxon signed-rank test for non-normally distributed data. To establish a physiological baseline for identifying postinjury pathological alterations, normal reference intervals (2.5^th^–97.5^th^ percentiles) along with the median and its bootstrap 95% confidence interval (CI) were calculated for each D-ULM feature using data from 30 healthy rats. Differences among the three injury phenotypes were assessed using one-way ANOVA with Tukey’s post-hoc test for normally distributed continuous variables, or the Kruskal—Wallis test with Dunn’s post-hoc test for non-normally distributed variables. Correlations between the D3 conventional ULM or D-ULM features and the D14 histopathological CAPs were evaluated using Spearman’s rank correlation coefficient, with statistical significance determined following false discovery rate (FDR) correction for multiple comparisons. A two-sided *P* < 0.05 was considered statistically significant.

### AI statement

DeepSeek (V4.0) was solely used for language polishing and grammar checking. No AI tools were used for data collection, data analysis, figure generation, or the conceptualization and interpretation of the research findings.

## Results

### Noninvasive ULM reveals endometrial microvasculature in rats

**Figure 2:**
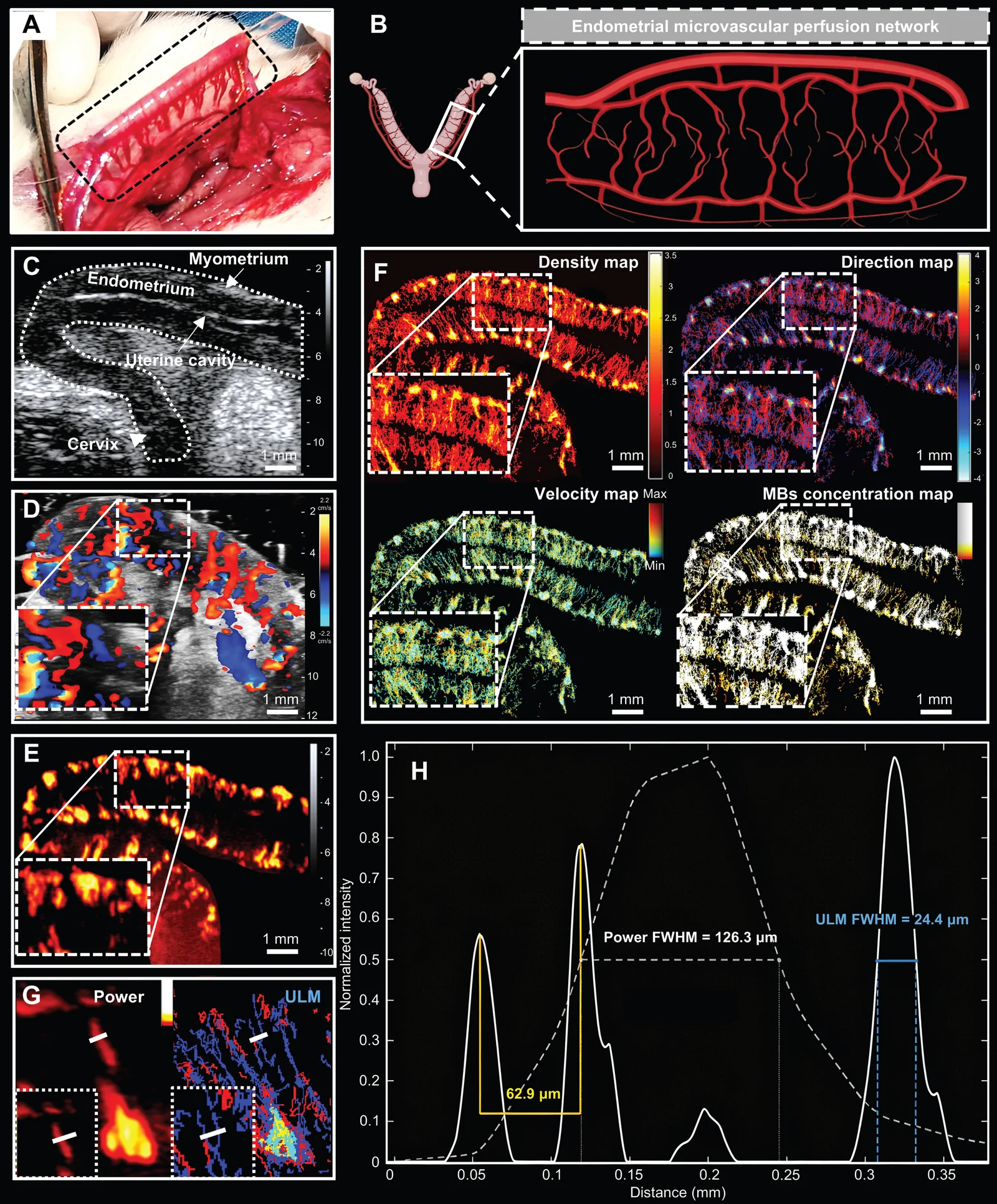
** Multimodal ultrasound images of endometrial microvasculature in rats.** (A, B) Anatomical example diagrams and schematic illustrations of the endometrial microvascular perfusion network in rats. (C) B-mode ultrasound. (D) Color Doppler flow imaging. (E) Power Doppler imaging. (F) ULM imaging, including density map, direction map, velocity map, and microbubble concentration map. (G, H) Intensity as a function of the lateral cross-section of the segmented vessel. The FWHM values for power Doppler and ULM are 126.3 μm and 24.4 μm, respectively. MBs: Microbubbles. Scale bar: 1 mm.

Achieving high-resolution, noninvasive imaging of the endometrial microvascular network remains a major challenge in reproductive medicine. The endometrial microvasculature originates from the main uterine arteries, branches into arcuate arteries and first-order branches, and culminates in microvessels with diameters of only a few micrometres—dimensions well below the resolution limit of conventional ultrasound imaging (**Figure 2A-B**). In this study, the visualization of the rat endometrial microvascular network was compared among multimodal ultrasound modalities. The results show that CDFI has poor resolution for detecting tiny blood flows, exhibiting obvious color flow overflow artifacts that make it difficult to delineate vascular morphology (**Figure 2C-D**). Power Doppler revealed blood flow within the endometrium, but the information it provided was limited, consisting predominantly of sparse flow signals in the first branch of the uterine arteries (**Figure 2E**). Leveraging the quantitative vascular parameters and high-resolution analytical capacity of ULM, we achieved high-definition ultrasound imaging of transmural microperfusion in the rat endometrium without invasive intrauterine procedures. As shown in **Figure 2F**, ULM resolved complex microvascular network structures consisting of arterioles and venules within the uterus and endometrium. Based on ULM images, the endometrial microvasculature exhibited a slight initial increase followed by a decline in branch density and vessel diameter from the myometrial side toward the uterine cavity. As measured by full width at half maximum (FWHM), ULM clearly distinguished vessels as small as 24.4 µm from background noise, representing an approximately 5.2-fold resolution enhancement over power Doppler and substantially improving the visualization of the entire spiral artery network of the endometrium (**Figure 2G-H**).

### ULM captures depth-dependent endometrial microvascular layering in normal rats

**Figure 3:**
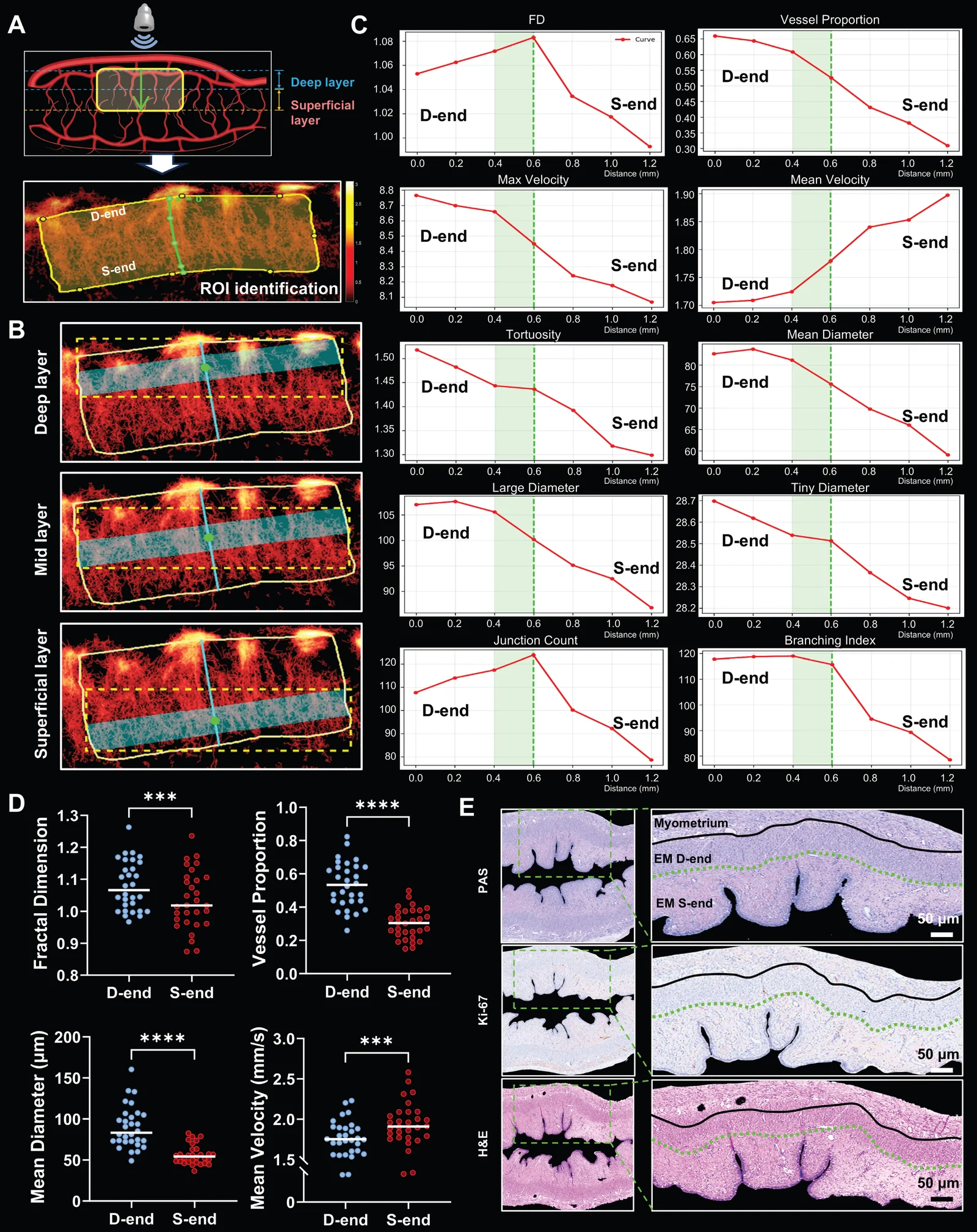
** Depth-dependent vascular layering in the normal endometrium revealed by ULM imaging.** (A, B) Schematic illustration of stepwise scanning from the myometrial (deep layer) side to the uterine cavity (superficial layer) side. (C) Representative depth-profile curves of ten conventional ULM parameters, including FD, vessel proportion, max velocity, mean velocity, tortuosity, mean diameter, large/tiny diameter, junction count, and branching index. Of note, the portions of the curves exceeding 1 mm should be interpreted with caution due to potential delineation errors. (D) Comparison of ULM parameters between the deep and superficial layers (n = 30). Statistical significance was determined by paired t test (****P < 0.0001, ***P < 0.001, **P < 0.01). (E) Histological validation at the transitional zone (relative depth 40–60%) using PAS, Ki-67, and H&E staining. Scale bar: 50 μm.

To systematically characterize the normal endometrial microvasculature and identify potential functional boundaries, we scanned the endometrium from the myometrial side to the uterine cavity side in equal steps (**Figure 3A-B**). Ten conventional ULM parameters were plotted as depth-profile curves (**Figure 3C**). The parameters exhibited depth-dependent patterns with a notable transition zone within the relative depth range of 40–60%. Specifically, FD and junction count initially increased and then decreased in most rats (26/30), whereas vessel proportion, maximum velocity, tortuosity, mean diameter, large diameter, tiny diameter and branching index showed a downward trend from the deep layer toward the superficial layer. In contrast, the mean velocity displayed the opposite trend. Statistical comparison between the deep and superficial layers confirmed significant differences in FD (*P* < 0.01), vessel proportion (*P* < 0.001), mean diameter (*P* < 0.001), and mean velocity (*P* < 0.01) (**Figure 3D**). The physiological depth-dependent variations identified by ULM have been verified by PAS staining, Ki-67 immunohistochemistry, and H&E staining, which revealed differences in metabolism, cell proliferation, and anatomical structure between the deep and superficial layers (**Figure 3E**). From the standpoint of histology, the region lying between 40-60% of relative depth is transitional, with Ki-67-positive cells significantly more numerous in the superficial layer (*P* < 0.05), an observation compatible with rapid functionalis proliferation. Collectively, these observations confirm that ULM describes layer-specific vascular and functional features of the normal endometrium.

### Normal reference interval of D-ULM features reveals depth-dependent physiological characteristics of the endometrial microvasculature

**Figure 4:**
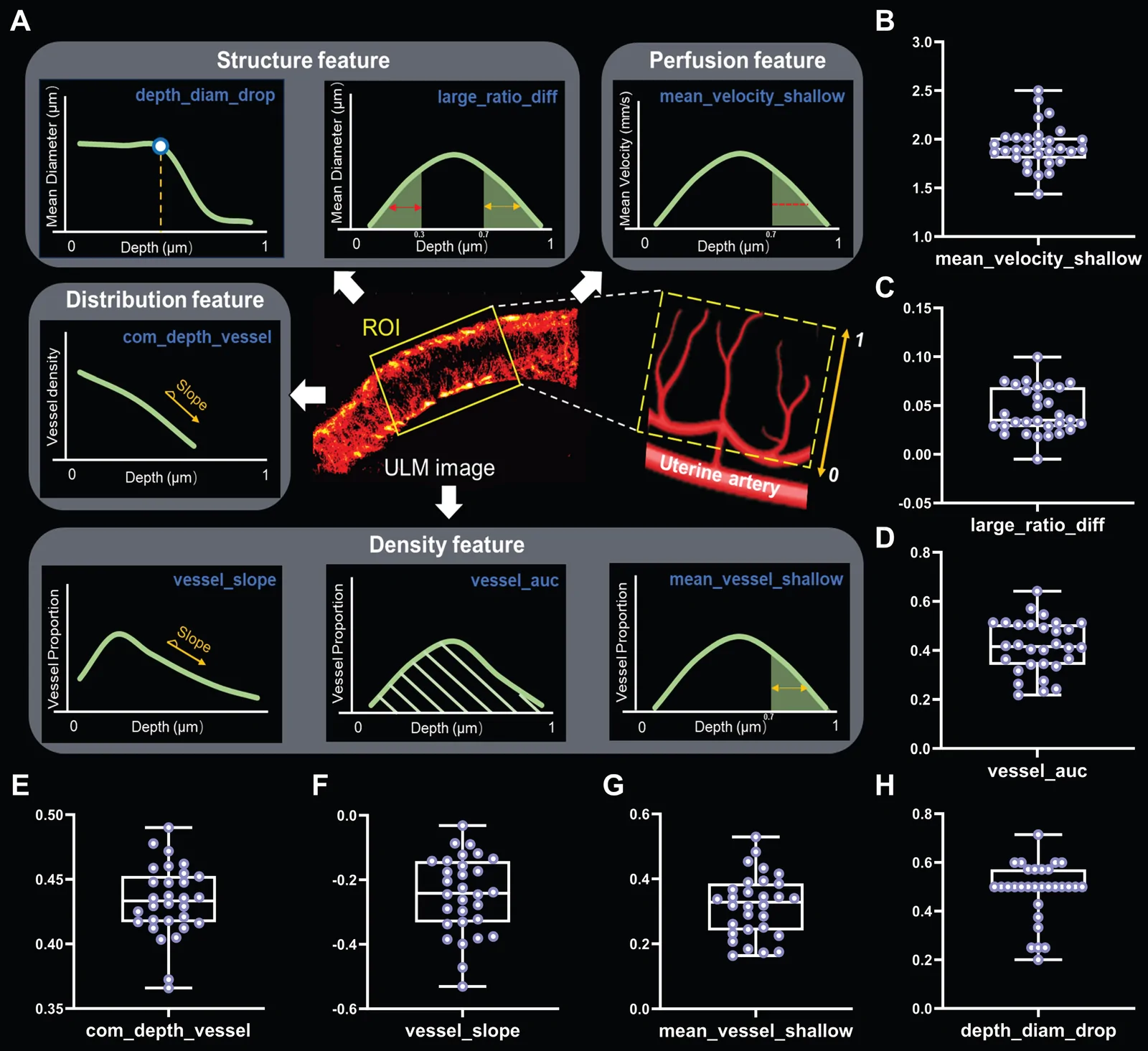
** Normal reference intervals of D-ULM features.** (A) Schematic illustration defining the seven D-ULM features. (B–H) Reference intervals (2.5^th^–97.5^th^ percentiles) and medians (with bootstrap 95% CI) for each feature (n = 30).

Building on the ability of ULM to resolve depth-dependent endometrial vascular architecture, we extracted seven depth-derived features from normal rats and defined them as D-ULM parameters, which reflect depth-dependent microvascular structure, perfusion, distribution, and density (**Figure 4**). The median (with 95% confidence interval), normal range, and detailed interpretation of each D-ULM feature are provided in Table 2. The results depict the depth-dependent anatomical characteristics of endometrial microperfusion in normal rats. The medians of com_depth_vessel and depth_diam_drop were 0.4 and 0.5, respectively, indicating that the vascular distribution center, as well as the transition zone of vascular types, resides at approximately 40–50% of endometrial depth. The large_ratio_diff was consistently positive, and together with a depth_diam_drop of approximately 0.5, this indicates that the proportion of large vessels is higher in the deep layer than in the superficial layer, with the transition occurring predominantly at mid-depth. Moreover, the vessel_slope was consistently negative, indicating a gradual decrease in vessel density from the deep layer to the superficial layer.

### D-ULM based unsupervised hierarchical clustering identifies endometrial injury-recovery phenotypes

**Figure 5:**
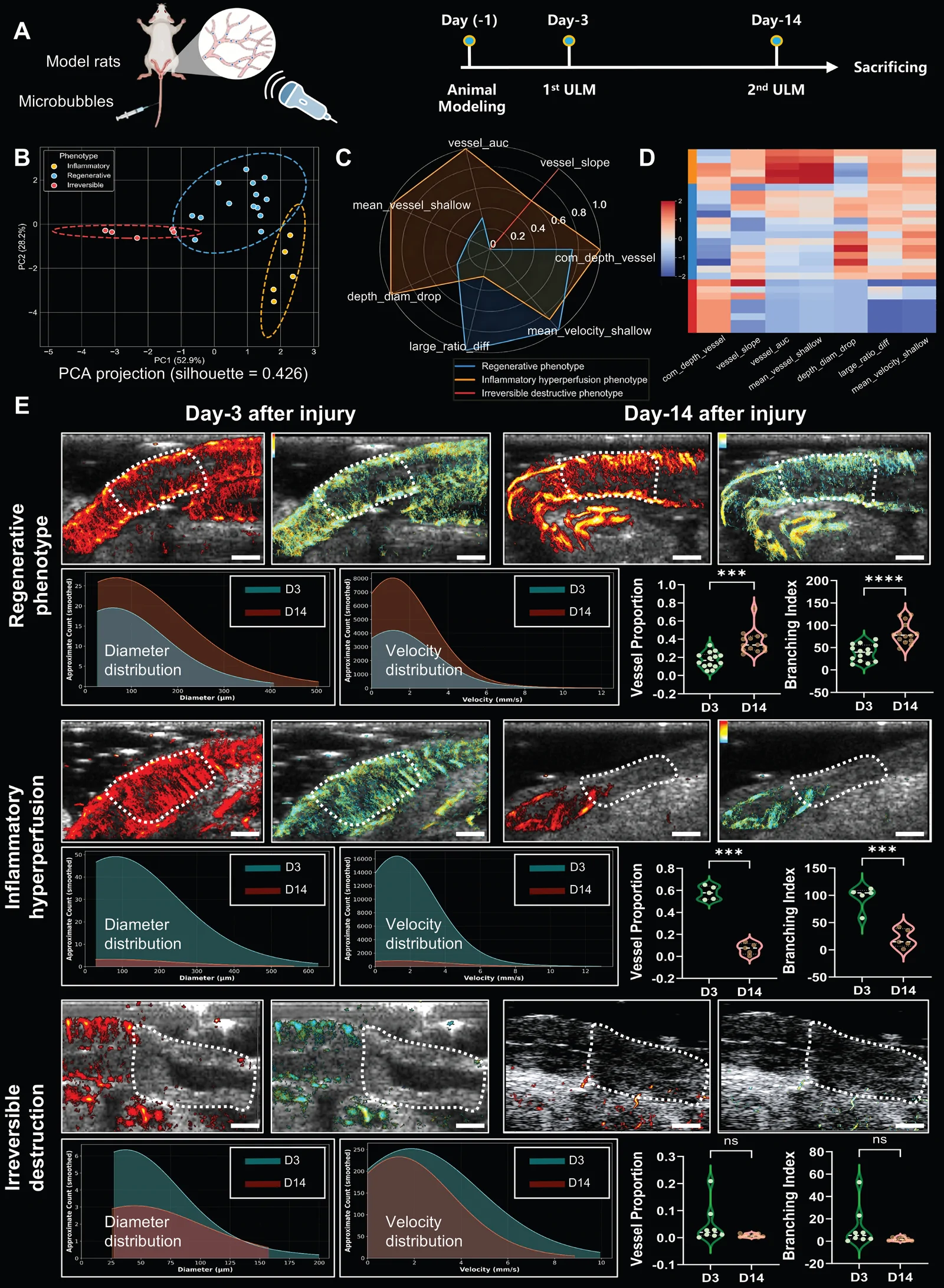
** Unsupervised clustering of day-3 D-ULM features identifies three injury phenotypes.** (A) Schematic timeline of the experiment. (B) PCA projection of the three phenotypes. (C) Radar chart of normalized features for each phenotype. (D) Feature heatmap of the three phenotypes. (E) Representative ULM density and velocity maps for each phenotype, along with curves of diameter and velocity of day 3 and day 14. Quantitative comparison of vessel proportion and branching index between day 3 and day 14 (n = 14 for Reg, n = 5 for IH, and n = 8 for ID). Statistical significance was determined by Kruskal-Wallis one-way ANOVA followed by Tukey’s post hoc correction (****P < 0.0001, ***P < 0.001, ns: not significant). Scale bar: 1 mm.

Building on the physiological baseline, we next characterized the heterogeneity of microvascular responses after injury in a data-driven manner. Unsupervised hierarchical clustering of day-3 D-ULM features was applied to 27 injured rats (3 rats were excluded due to accidental death), with the same rats followed longitudinally until day 14 (**Figure 5A**). Based on the D-ULM features, this analysis identified three distinct clusters (silhouette coefficient = 0.426, Supplementary Table S1). Bootstrap resampling (1,000 iterations) with replacement was performed to assess the robustness of this three-cluster solution. The mean adjusted Rand index between the bootstrap-derived clustering and the expert-based visual category was 0.790, and the average consistency per sample (the proportion of bootstrap replicates in which each rat was assigned to the same cluster as in the expert-based visual category) was 0.776, indicating substantial stability of the three-cluster solution relative to the external reference. As shown in Supplementary Figure S1, the collinearity among the depth-derived features did not undermine the robustness of the original clustering. The regenerative (Reg) phenotype tended to have moderate feature values and to show gradual vascular normalization over time; the inflammatory hyperperfusion (IH) phenotype showed high shallow-zone vessel proportion and velocity; and the irreversible destructive (ID) phenotype was marked by little or no signal for any feature. PCA projection (**Figure 5B**) reveals a distinct separation of the three phenotypes. The radar chart (**Figure 5C**) illustrates the normalized feature profiles, while the feature heatmap (**Figure 5D**) confirmes clustering consistency.

To preliminarily assess whether day-3 D-ULM clustering could provide insight into mid-to-long-term blood flow recovery after injury, we compared day-3 and day-14 vascular parameters. Representative ULM density and velocity maps for each phenotype are shown in **Figure 5E**. Diameter contribution and velocity contribution curves from day 3 to day 14 showed that the regenerative phenotype displayed a trend toward vascular normalization over time, whereas the irreversible destructive and inflammatory hyperperfusion phenotypes did not. Analysis of two representative features, vessel proportion (reflecting vascular density) and branching index (reflecting network complexity), revealed significant interphenotype differences at both day 3 and day 14. The Reg phenotype showed the most pronounced improvement, the ID phenotype maintained persistently low values, and the IH phenotype exhibited continuous deterioration.

Table 3 gives a summary of the D3 D-ULM features relevant to each phenotype. For the ID phenotype, almost every vascular measurement was close to zero, which implies very little or no detectable microvascular activity. The Reg phenotype had distinct yet moderate D-ULM characteristics, which suggest a partial return to normal microvascular function. By contrast, the IH phenotype showed large increments in overall perfusion, as shown by vessel_auc (0.743 [0.663, 0.766]). Notably, this phenotype showed an increasing trend in vascular density from the deep layer to the superficial layer. Mean_vessel_shallow (which measures superficial layer density) was strongly increased (0.702 [0.572, 0.768]) and ranked first among all phenotypes. Moreover, large_ratio_diff (0.018 [-0.006, 0.052]) was lower than that of the Reg phenotype (0.062 [0.023, 0.078]), which indicates a smaller variation in large vessel numbers between the two layers.

**Figure 6:**
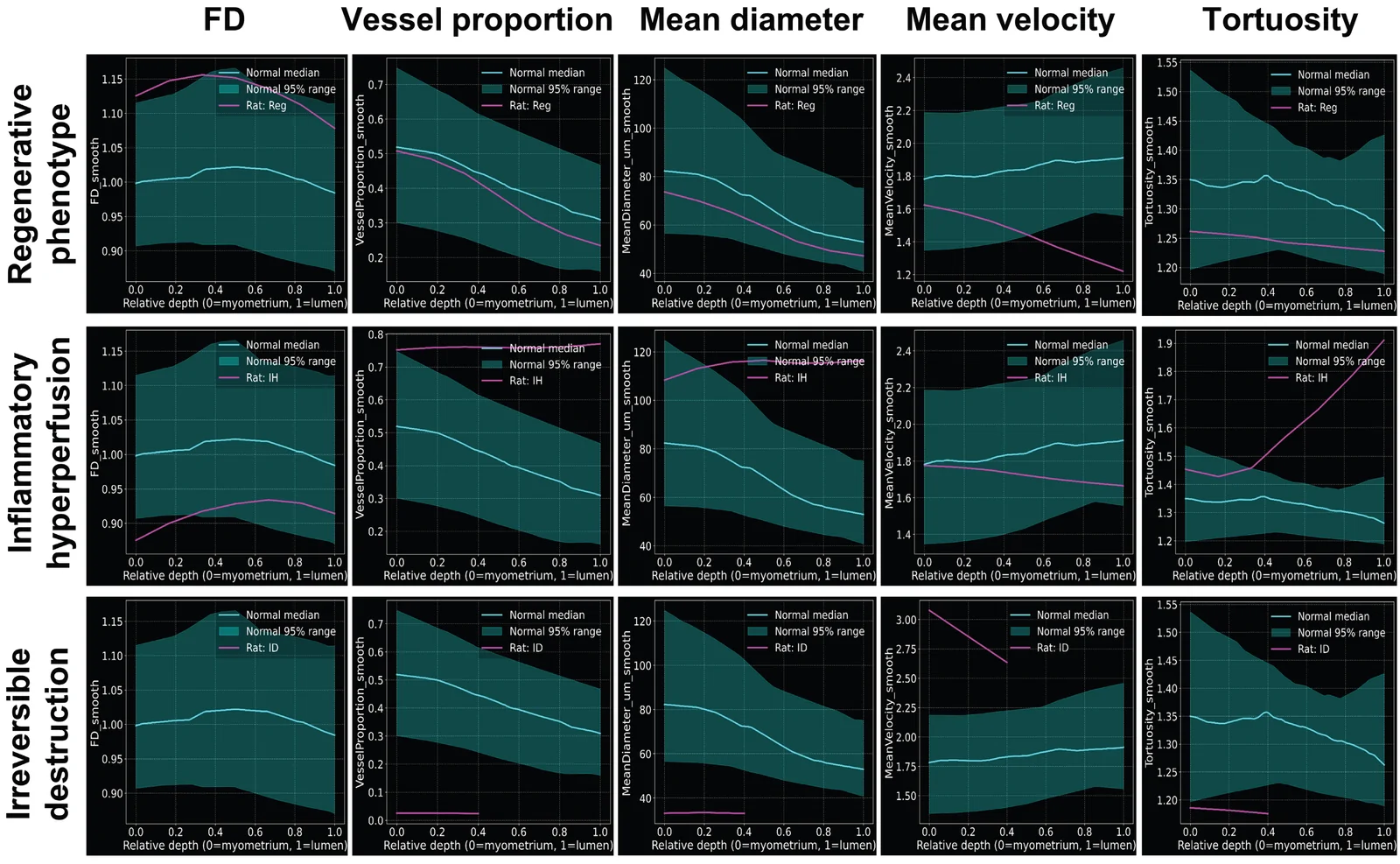
** Depth profiles of injury phenotypes versus normal reference.** Green curves (light green shading) represent the normal reference band (median ± IQR). Red curves indicate representative cases for each phenotype.

**Figure 6** presents representative depth profiles for each phenotype overlaid with the normal reference band. In the Reg phenotype, the day-3 vessel proportion curve fell largely within the normal reference band, particularly in the deep zone (close to the myometrial side). In the IH phenotype, the shallow-zone vessel proportion exceeded the upper reference limit, with both vessel proportion and mean velocity remaining consistently elevated. Additionally, tortuosity was increased in the superficial zone, further supporting a dysfunctional hyperemic response. In contrast, the curve of the ID phenotype was fragmented and discontinuous, largely falling outside the normal reference band, reflecting extensive vascular loss.

### Early D-ULM features and derived clusters reflect distinct histological fibrotic outcomes

**Figure 7:**
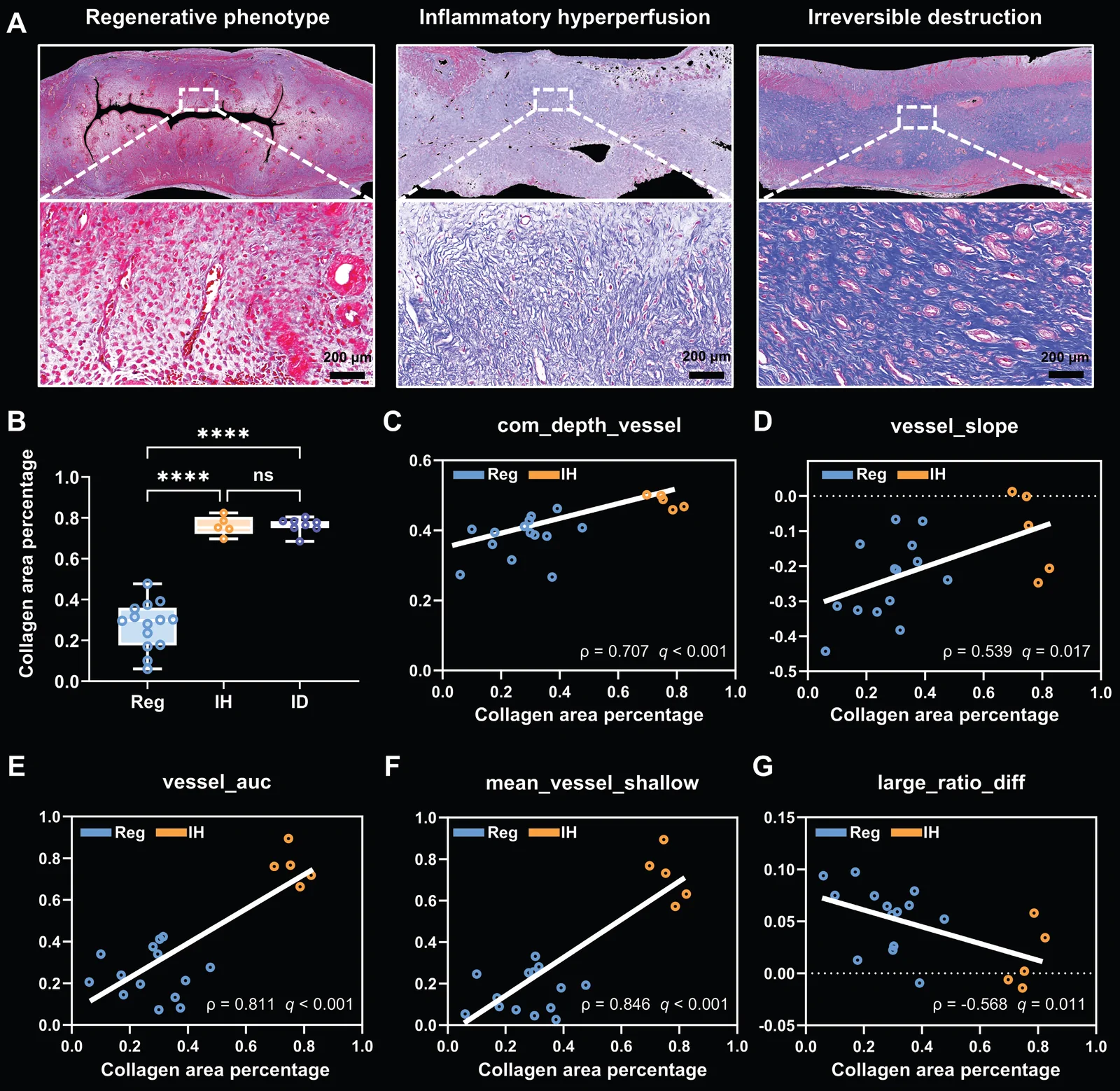
** Correlation analysis of early D-ULM features and derived clusters with late fibrotic outcome from pathological images.** (A) Representative Masson’s trichrome staining images of the three phenotypes. Scale bar: 200 μm. (B) Quantitative analysis of CAP among the three phenotypes (n = 14 for Reg, n = 5 for IH, and n = 8 for ID). (C-G) Spearman correlations between day-3 depth-derived features (com_depth_vessel, vessel_slope, vessel_auc, mean_vessel_shallow, large_ratio_diff) and day-14 CAP. Statistical significance was determined by Kruskal-Wallis one-way ANOVA followed by Dunn’s post hoc correction (****P < 0.0001, ns: not significant). Reg: regenerative phenotype, IH: inflammatory hyperperfusion phenotype, ID: irreversible destructive phenotype.

Subsequently, we performed a correlation analysis between D-ULM features and the quantified fibrosis area, measured as CAP by Masson staining on day 14. Representative images are presented for the three D-ULM derived clusters (**Figure 7A**). As shown in **Figure 7B**, post hoc Dunn tests revealed that the quantitative CAP values of the Reg phenotype (median 0.298, IQR 0.176–0.361) were significantly lower than those of the other two phenotypes (ID: median 0.772, IQR 0.750–0.784; IH: median 0.753, IQR 0.722–0.805). To avoid confounding the assessment of graded associations, correlation analyses between D-ULM features and CAP were performed only within the Reg and IH phenotypes, as the ID phenotype exhibited near-zero vascular signals across most features. A sensitivity analysis including all three phenotypes is provided in Supplementary Figure S2. After FDR correction, five D-ULM features remained significant (**Figure 7C–G**). Early com_depth_vessel (ρ = 0.707, 0.372 to 0.879), vessel_slope (ρ = 0.539, 0.097 to 0.803), vessel_auc (ρ = 0.811, 0.566 to 0.925), and mean_vessel_shallow (ρ = 0.846, 0.635 to 0.939) were positively correlated with day 14 fibrosis, whereas large_ratio_diff was inversely correlated (ρ = -0.568, -0.812 to -0.153). In addition, in the comparison between day-3 and day-14 D-ULM features, vessel_auc and mean_vessel_shallow were significantly increased in the Reg phenotype, whereas they were markedly decreased in the IH phenotype (Supplementary Figure S3). Of note, conventional ROI-based ULM parameters (|ρ| < 0.6, Supplementary Table S2) showed weaker correlations with fibrosis area than the depth derived features, underscoring the value of preserving depth information. Collectively, these findings establish a close correspondence between early D-ULM based features (both individual- and cluster-derived) and the extent of late fibrotic remodeling, validating their potential as imaging indicators of postinjury repair.

### Microvascular abnormalities are associated with immune microenvironment imbalance after endometrial injury

**Figure 8:**
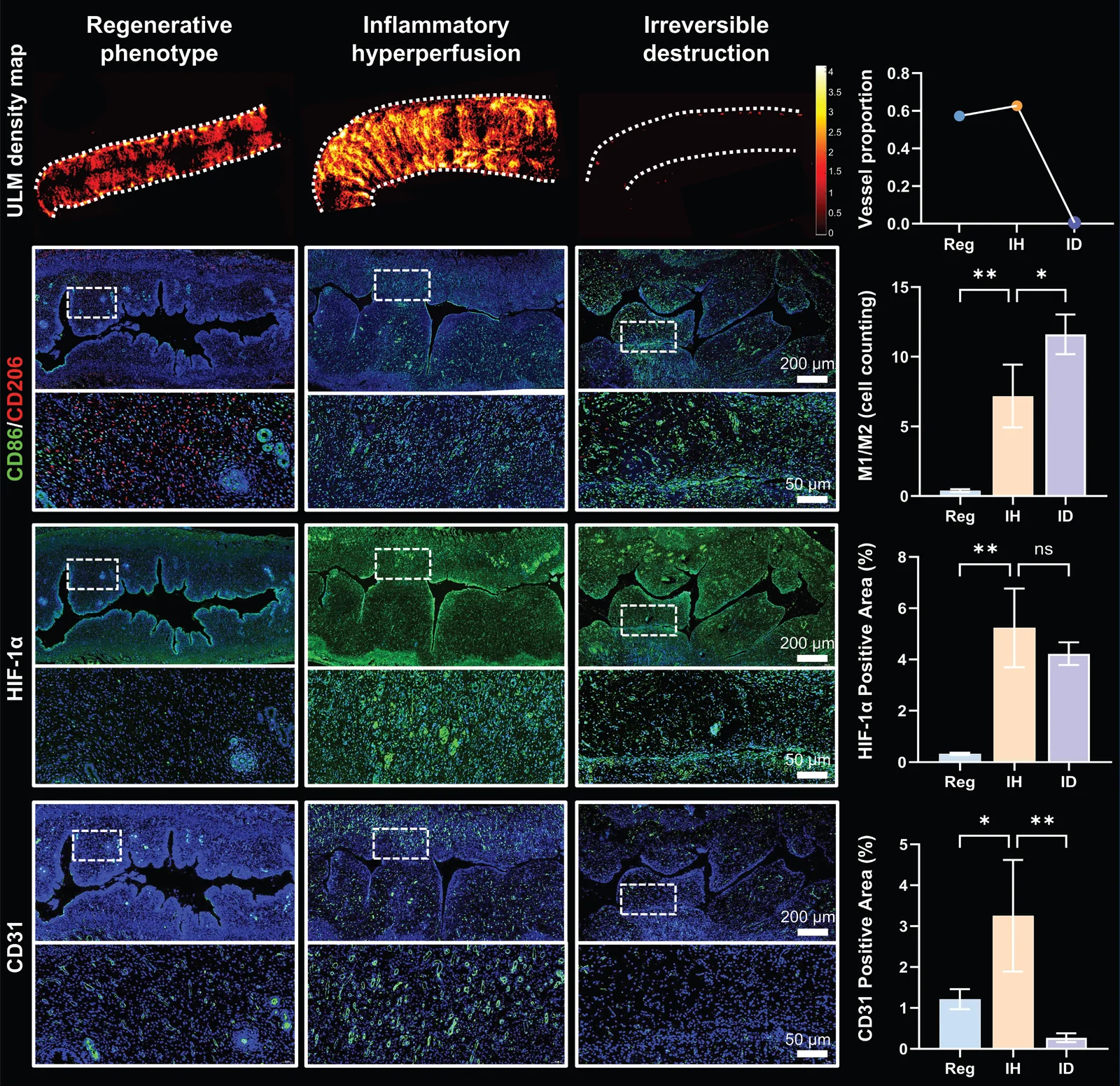
** Immunofluorescence staining of D3 D-ULM phenotype-specific microenvironments.** Representative IF images and quantification of macrophage polarization (M1: iNOS, M2: CD206), hypoxia (HIF-1α) and angiogenesis (CD31) in three D-ULM phenotypes (n = 3). Statistical significance was determined by Kruskal-Wallis one-way ANOVA followed by Tukey’s post hoc correction (**P < 0.01, *P < 0.05, ns: not significant). Scale bars: 200 μm (low magnification) and 50 μm (high magnification).

Finally, we investigated whether the observed changes in D-ULM-derived clusters corresponded to alterations in the immune microenvironment, as assessed by pathological images. To determine whether microvascular abnormalities were associated with postinjury immune microenvironment imbalance, IF staining was performed for HIF-1α (hypoxia marker), CD31 (angiogenesis marker), and macrophage polarization markers (CD86 for M1, CD206 for M2) across the different D-ULM phenotypes, and the expression levels were quantitatively compared (**Figure 8**). Distinct IF patterns emerged among the three phenotypes. The IH phenotype exhibited marked CD31 expression, indicating reactive microvascular proliferation, accompanied by appreciable HIF-1α positivity and a much higher M1/M2 ratio than the Reg phenotype (*P* < 0.01), a pattern consistent with hypoxia-induced pro-inflammation. The Reg phenotype had moderate CD31 elevation, almost normal HIF-1α values, and an immune microenvironment dominated by M2 macrophages, a condition closely resembling that of the normal endometrium. In contrast, the ID phenotype was marked by pronounced endometrial hypoxia (signaled by HIF-1α staining), a marked shift toward an M1-dominated phenotype, and low CD31 levels, a pattern suggestive of persistent inflammation with impaired angiogenesis. These findings collectively support the view that D-ULM-derived clustering reflects interindividual immune microenvironmental differences postinjury, potentially linking early D-ULM states to later fibrosis.

## Discussion

Early assessment of endometrial fibrosis and functional recovery after injury remains challenging due to the lack of high-resolution microvascular imaging techniques and compatible analytical methods. Although ULM provides noninvasive visualization of microvascular structure and function, offering a promising solution, conventional quantitative ULM studies rely on ROI-based global averaging, which fails to capture depth-dependent information essential for understanding the physiological and pathological changes of the endometrium. In this study, we pioneered a depth-derived feature analysis for endometrial ULM imaging, termed D-ULM, and established a normal D-ULM reference interval based on vascular functional gradients, thereby delineating the depth-resolved microperfusion landscape of the normal rat endometrium. Through unsupervised clustering, we identified three vascular phenotypes with significantly different prognostic outcomes in the early postinjury phase: the regenerative phenotype, the inflammatory hyperperfusion phenotype, and the irreversible destructive phenotype. Our findings indicate that these D-ULM-based phenotypes are significantly correlated with later postinjury blood flow recovery and the severity of fibrosis. Collectively, these results introduce a noninvasive, stratified, and prognostic imaging paradigm for the endometrial microvasculature, offering a high-resolution quantitative method to inform individualized therapeutic decisions and may help prevent irreversible fibrosis following endometrial injury.

An important finding of this study is the establishment of a new, quantifiable functional boundary of the endometrial microvasculature, based on depth-derived ULM. Analyzing vascular structure at specific depths is crucial for understanding both physiological function and disease; for example, OCT angiography has been applied to retinal disease to separate the superficial and deep capillaries 19. Regarding endometrial fibrosis, damage to the basal layer has been considered a central determinant and an independent indicator of later fibrosis progression—a view supported by substantial clinical evidence 20,21. Nevertheless, no currently available imaging technique can non-invasively visualize the boundary between the basal and functional layers. Regarding normal physiology, the basal and functional layers (which are at separate levels within the endometrium) differ in structure and in function 22. Recent work based on single-cell transcriptomics indicated the heterogeneity among the epithelial and stromal elements of the endometrium, clarifying the complexity of this tissue 23. From the viewpoint of histology, the present discussion of the differences between these two layers is primarily concerned with cell density, glandular form, vascular anatomy, and cyclic variation. Regarding the endometrial microvasculature, the spiral arteries (less than 100 μm in diameter) adjacent to the uterine cavity pass through the functional layer in a complicated, tortuous pattern, which facilitates cyclic shedding and embryo implantation. By contrast, the vessels of the basal layer are larger and constitute a dense network, giving rise to regenerative ability and basal vascular stability 24. Even so, current imaging methods remain limited in fully depicting the endometrial microvasculature due to resolution limitations. The present work has demonstrated the utility of ULM for visualizing endometrial microperfusion and has established depth-profile curves of standard ULM parameters. These curves reveal a distinct transition zone (0.4–0.6 relative depth) characterized by marked contrasts in vascular features, flow speed, and morphology between the superficial and deep layers. Compared with the deep layer, the superficial layer has a smaller vessel proportion, lower maximal velocity, smaller vessel diameter, and reduced tortuosity and complexity (lower fractal dimension and branching index). These findings coincide with known features of spiral arteries in the functional layer, and are consistent with the regional division indicated by histopathological examination.

The use of tissue-specific properties in quantitative imaging is a novel strategy that helps accelerate the clinical translation of these imaging methods 25. Most prior work with ULM has concentrated on region-specific averages of parameters such as vascular density or flow velocity 26,27. Given the dependence of endometrial fibrosis on injury depth and the capacity of ULM for high-resolution deep-tissue imaging, we devised D-ULM to assess depth-related vascular parameters and to clarify the relationship between early postinjury microperfusion and later fibrosis. Here, in addition to conventional ULM features, we included junction count and branching index to more accurately characterize the morphology of endometrial microperfusion 28. Notably, most of the D-ULM features that reflect depth-related changes show a stronger correlation with fibrotic outcomes than ROI-based ULM metrics. Among these D-ULM features, an increase in com_depth_vessel was associated with a shift of the microvascular distribution center toward the uterine cavity—an effect particularly marked in the IH phenotype, which reflected increased superficial microvessels and was consistent with elevated mean_vessel_shallow. In addition, vessel_slope, which quantifies the decline in vessel density from the deep to the superficial layer, was positively associated with fibrosis severity. A less negative (flattened) slope reflects a diminished density gradient between the two layers, indicating loss of the physiological functional zonation of the endometrium. This disruption of depth-dependent vascular heterogeneity was closely linked to postinjury fibrotic outcomes. Mechanistically, a flattened slope may reflect either aberrant superficial neovascularization driven by inflammation (as seen in the IH phenotype) or profound deep zone vascular destruction (as in the ID phenotype), both of which could compromise regenerative capacity and promote fibrotic remodeling. Similarly, the negative correlation between large_ratio_diff and fibrosis severity suggests that loss of the deep to shallow gradient in large vessel proportion, whether due to deep vascular destruction or superficial large vessel neogenesis, is associated with worse fibrotic outcomes. In addition, we observed a continuous gentle increase in the mean velocity from the deep layer to the superficial layer in normal rats. In the deep zone, high velocity flow in spiral arteries coexists with slow moving blood near vessel walls; in the superficial zone, the capillary network eliminates extreme highs and stagnation, resulting in more homogeneous perfusion and a higher mean velocity. This transition from a laminar pattern to a uniform slow-flow pattern may reflect the physiological adaptation for efficient microcirculatory exchange in the functional layer.

Ethanol exposure duration was generally associated with fibrosis severity, but the relationship was not strictly dose-dependent, given the marked individual heterogeneity in postinjury fibrotic outcomes 29. This was confirmed in our study: while severe fibrosis was observed in 44% (4/9) of rats in the severe injury group, it occurred in 60% (6/10) of rats in the mild injury group (Supplementary Figure S4). To better correlate early vascular features with late fibrotic outcomes, we performed unsupervised hierarchical clustering of day-3 D-ULM features and identified three distinct microvascular response phenotypes: Reg (preserved deep-zone vessels, near-normal depth profiles), IH (elevated superficial-zone vessel proportion and velocity, suggestive of hypoxia-driven hyperemia), and ID (pan-endometrial vascular collapse with global perfusion loss). Although correlation analyses between individual D-ULM features and immunofluorescence markers were not performed, consistent patterns emerged within each phenotype. In the IH phenotype, elevated mean_vessel_shallow and vessel_auc, together with high HIF-1α and CD31 expression, suggest hypoxia-driven reactive angiogenesis. In the ID phenotype, reduced com_depth_vessel and large_ratio_diff, accompanied by diminished CD31, point to impaired vascular regeneration and tissue ischemia. The early postinjury microenvironment is characterized by the initiation of M1-to-M2 macrophage polarization, a transient surge in HIF-1α following acute tissue damage, and a dynamic balance between endothelial injury and early CD31-mediated angiogenic repair 30,31. However, the eventual outcomes of this microenvironment diverge depending on the severity and persistence of the insult. Both the IH and ID phenotypes lead to severe fibrosis, but through different mechanisms. In the IH phenotype, an ongoing inflammatory stimulus leads to HIF-1α-mediated M1 inflammation and abnormal angiogenesis, which fail to relieve tissue hypoxia even when perfusion is high. By contrast, for the ID phenotype, the injury is severe enough to cause major endothelial damage and marked tissue hypoxia, along with microvascular destruction, impaired angiogenesis, and global hypoperfusion 32,33. These distinct mechanisms explain both the intense superficial hyperemia characteristic of the IH phenotype and the near-absence of microvascular signals in ID phenotype. From a clinical translation perspective, day-3 D-ULM clustering may help identify patients at high risk of fibrosis, thereby enabling early intervention. Patients with the IH phenotype may benefit from anti-inflammatory therapy 34, whereas those with the ID phenotype may be suitable for stem-cell therapy 35. Although separate pathogenic mechanisms support early D-ULM in distinguishing the two high-risk phenotypes and planning individualized therapy 36, such therapies still need to be tested in future interventional trials.

The current animal experiments required surgical exposure of the uterine horns to achieve optimal image quality. This surgical procedure relates to the preclinical imaging setup rather than the imaging technology itself, as clinical ULM is performed with external transducer without intrauterine insertion, offering noninvasive imaging with resolution comparable to intra-cavitary techniques (e.g., photoacoustic endoscopy). Despite these translational advantages, the current study has several limitations that should be acknowledged. First, further validation of this paradigm in a disease treatment setting is lacking. Second, tissue distortion during histological preparation, a common challenge, may affect location matching 7. We have not yet achieved absolute registration between ULM images and pathological sections. Third, although ULM provides high resolution in two-dimensional acquisition, it does not capture the full three-dimensional configuration of the endometrial vasculature. This limitation might be overcome by three-dimensional ULM, which would help to reconstruct the tissue’s microvascular pattern more completely 37. Fourth, due to the limited sample size, the stability of the clustering should be interpreted with caution; moreover, the present analyses are limited to correlations and do not involve predictive models or external validation. Nevertheless, candidate D-ULM features, such as shallow zone vessel proportion (mean_vessel_shallow) and vascular distribution center (com_depth_vessel), showed significant associations with fibrosis severity and hold promise as potential inputs for future predictive models. As transvaginal ULM techniques continue to advance, the broad applicability and stability of D-ULM clusters will need to be confirmed through multicenter, large-scale clinical trials.

## Conclusion

In summary, this study establishes a depth-based ULM framework for noninvasive, repeatable, high-resolution imaging of the endometrial microvasculature. In the normal group, we extracted depth-dependent vascular data and formed a normal reference band to characterize the physiology of depth-resolved microperfusion in the endometrium. In the injury groups, unsupervised clustering of early D-ULM features identified three distinct microvascular phenotypes. The D-ULM properties of the phenotypes correlated well with the corresponding variations in the immune microenvironment and with subsequent fibrotic outcomes. These findings demonstrate that retention of depth-related information is critical both to the functional analysis of the endometrium and to the assessment of postinjury fibrosis risk. While further validation in larger cohorts and clinical settings is needed, the D-ULM framework offers an imaging tool that may aid in personalized treatment planning. It may help prevent irreversible fibrosis in the early postinjury stage, thereby enabling a transition from a diagnose-and-treat approach to a predict-and-prevent strategy.

## Supplementary Material

Supplementary figures and tables.

## Figures and Tables

**Table 1:** Hemodynamic and morphological features extracted from ULM images.

Feature	Significance
Fractal dimension	Box-counting-based measure of microvascular network complexity; higher values indicate more intricate, space-filling vascular architecture
Vascular proportion	Microbubble passage rate per pixel normalized by acquisition time, reflecting functional microvascular density and tissue blood volume
Max velocity (mm/s)	Peak or 95^th^ percentile of microbubble velocities, indicating maximal perfusion capacity and sensitivity to flow resistance
Mean velocity (mm/s)	Arithmetic mean of local microbubble velocities, serving as a surrogate for blood flow and perfusion efficiency
Tortuosity	Ratio of vessel segment actual length to endpoint distance
Mean diameter (μm)	Local thickness-derived lumen size, reflecting vessel caliber and vasomotor status
Large vessel diameter (μm)	The average diameter of vessels with a diameter that exceeds the 40 μm^*^ threshold
Tiny vessel diameter (μm)	The average diameter of vessels with a diameter below the 40 μm^*^ threshold
Junction count	The total number of vessel junctions in the ROIs
Branching index (mm⁻²)	Number of branching points per unit area (mm²), quantifying network connectivity and topological complexity

Note:^ *^Based on the distribution of vessel diameters in normal rats 18.

**Table 2:** Normal reference intervals of D-ULM features.

D-ULM features	Median (95% CI)	Normal range (2.5^th^–97.5^th^ %)	IQR	Significance
com_depth_vessel	0.433 (0.420–0.448)	0.371–0.481	0.034	Vascular distribution center located at ~43% endometrial depth
vessel_slope	-0.242 (-0.293–-0.175)	-0.488–-0.072	0.183	Negative value reflects decreasing vessel density from myometrium to uterine cavity
vessel_auc	0.416 (0.365–0.492)	0.229–0.590	0.164	Overall vascular abundance
mean_vessel_shallow	0.328 (0.261–0.352)	0.171–0.496	0.135	Quantifies superficial layer vascular density
depth_diam_drop	0.500 (0.500–0.500)	0.236–0.631	0.071	Approximately 0.5, marking the transition from large vessels to capillaries
large_ratio_diff	0.035 (0.030–0.056)	0.012–0.082	0.039	Consistently positive, indicating deeper zone large-vessel advantage
mean_velocity_shallow (mm/s)	1.897 (1.870–1.990)	1.577–2.431	0.194	Reflects functional layer perfusion level

Note: CI (confidence Interval), IQR (interquartile range)

**Table 3:** D-ULM features in different phenotypes.

D-ULM feature	Regenerative phenotype	Inflammatory hyperperfusion	Irreversible destruction
com_depth_vessel	0.394 (0.327, 0.425)	0.479 (0.459, 0.500)	0 (0, 0.137)
vessel_slope	-0.255 (-0.329, -0.150)	-0.145 (-0.239, -0.001)	0 (-0.024, 0)
vessel_auc	0.227 (0.158, 0.366)	0.743 (0.663, 0.766)	0 (0, 0.012)
mean_vessel_shallow	0.156 (0.059, 0.255)	0.702 (0.572, 0.768)	0 (0, 0.003)
depth_diam_drop	0.313 (0.036, 0.500)	0.500 (0.429, 0.500)	0.084 (0, 0.425)
large_ratio_diff	0.062 (0.023, 0.078)	0.018 (-0.006, 0.052)	0 (0, 0)
mean_velocity_shallow (mm/s)	1.829 (1.458, 2.044)	1.586 (1.386, 2.031)	0 (0, 0.397)

Note: Data are presented as the median (Q1, Q3). The zero median results from the predominance of zero values in this phenotype.

## Data Availability

Data are available from the corresponding author upon reasonable request.

## Abbreviations

ANOVA: analysis of variance
CAP: collagen area percentage
CI: confidence interval
D-ULM: depth-derived ultrasound localization microscopy
DAPI: 4’,6-diamidino-2-phenylindole
FD: fractal dimension
FDR: false discovery rate
FWHM: full width at half maximum
ID: irreversible destruction phenotype
IF: immunofluorescence
IH: inflammatory hyperperfusion phenotype
IQ: In-phase and Quadrature
IQR: interquartile range
IUA: intrauterine adhesions
PAS: periodic acid–Schiff
PCA: principal component analysis
Reg: regenerative phenotype
RF: radio frequency
ROI: regions of interest
SD: standard deviation
SVD: singular value decomposition
ULM: ultrasound localization microscopy
