# Supplementary material for: Mapping endometrial vascular functional gradients using depth-derived ultrasound localization microscopy (D-ULM) for early stratification of postinjury fibrotic remodeling and therapeutic guidance
Source: Theranostics. 2026 Aug 12;16(15):8669–88. doi: 10.7150/thno.138622 (PMC13549232; doi:10.7150/thno.138622)
Supplement: Supplementary file 1 [file thnov16p8669s1.pdf]

# Supplementary file

## Mapping endometrial vascular functional gradients using depth derived ultrasound localization microscopy (D-ULM) for early stratification of post-injury fibrotic remodeling and therapeutic guidance

Xiaowen Liang[1,2,3,6#], Le Gao[4,5#], Zhili Guo[2,3,6], Meng Du[2,3,6], Yue Pan[5], Yixiang Lian[7], Yu Qiang[5], Haijun Luo[7], Ying Zhang[2,3,6], Xiaoyan Kui[1*], Hairong Zheng[5*], Weibao Qiu[5*], Zhiyi Chen[2,3,6*]

[1] School of Computer Science and Engineering, Central South University, Changsha, 410083, China.
[2] Key Laboratory of Medical Imaging Precision Theranostics and Radiation Protection, College of Hunan Province, the Affiliated Changsha Central Hospital, Hengyang Medical School, University of South China, Changsha, 410004, China.
[3] Institute of Medical Imaging, Hengyang Medical School, University of South China, Hengyang, 410004, China.
[4] University of Chinese Academy of Sciences, Beijing, 101408, China.
[5] Shenzhen key laboratory of ultrasound imaging and therapy, State Key Laboratory of Biomedical Imaging Science and System, Shenzhen Institutes of Advanced Technology, Chinese Academy of Sciences, Shenzhen, 518055, China.
[6] Institute for Future Sciences, University of South China, Changsha, 410008, China.
[7] Department of Pathology, the Affiliated Changsha Central Hospital, Hengyang Medical School, University of South China, Changsha, 410004, China.

[#]These authors contributed equally to this work.
[*]Correspondence to: Xiaoyan Kui, xykui@csu.edu.cn
Hairong Zheng, hr.zheng@siat.ac.cn
Weibao Qiu, wb.qiu@siat.ac.cn
Zhiyi Chen, zhiyi_chen@usc.edu.cn

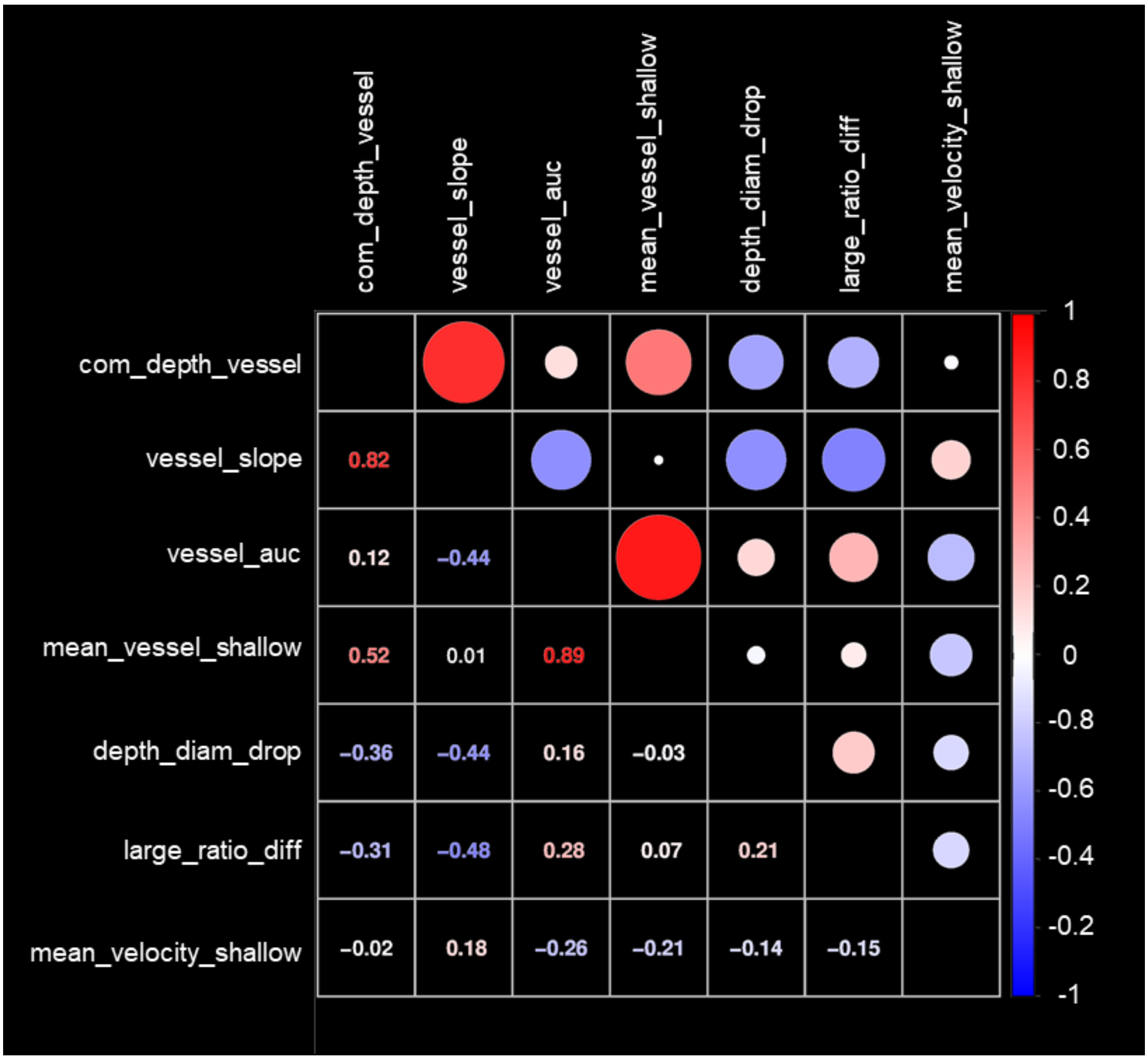


**Figure S1.** Correlation matrix of D-ULM features (multicollinearity analysis), demonstrating strong positive correlations between com_depth_vessel and vessel_slope, as well as between mean_vessel_shallow and vessel_auc. To assess the impact of collinearity, principal component analysis was performed and the first three principal components, explaining 90% of the total variance, were retained. Hierarchical clustering on the PC scores yielded a cluster assignment with an adjusted Rand index of 0.697 compared to the original clustering, indicating substantial agreement and demonstrating that the original clustering is robust to collinearity among the depth-derived features.

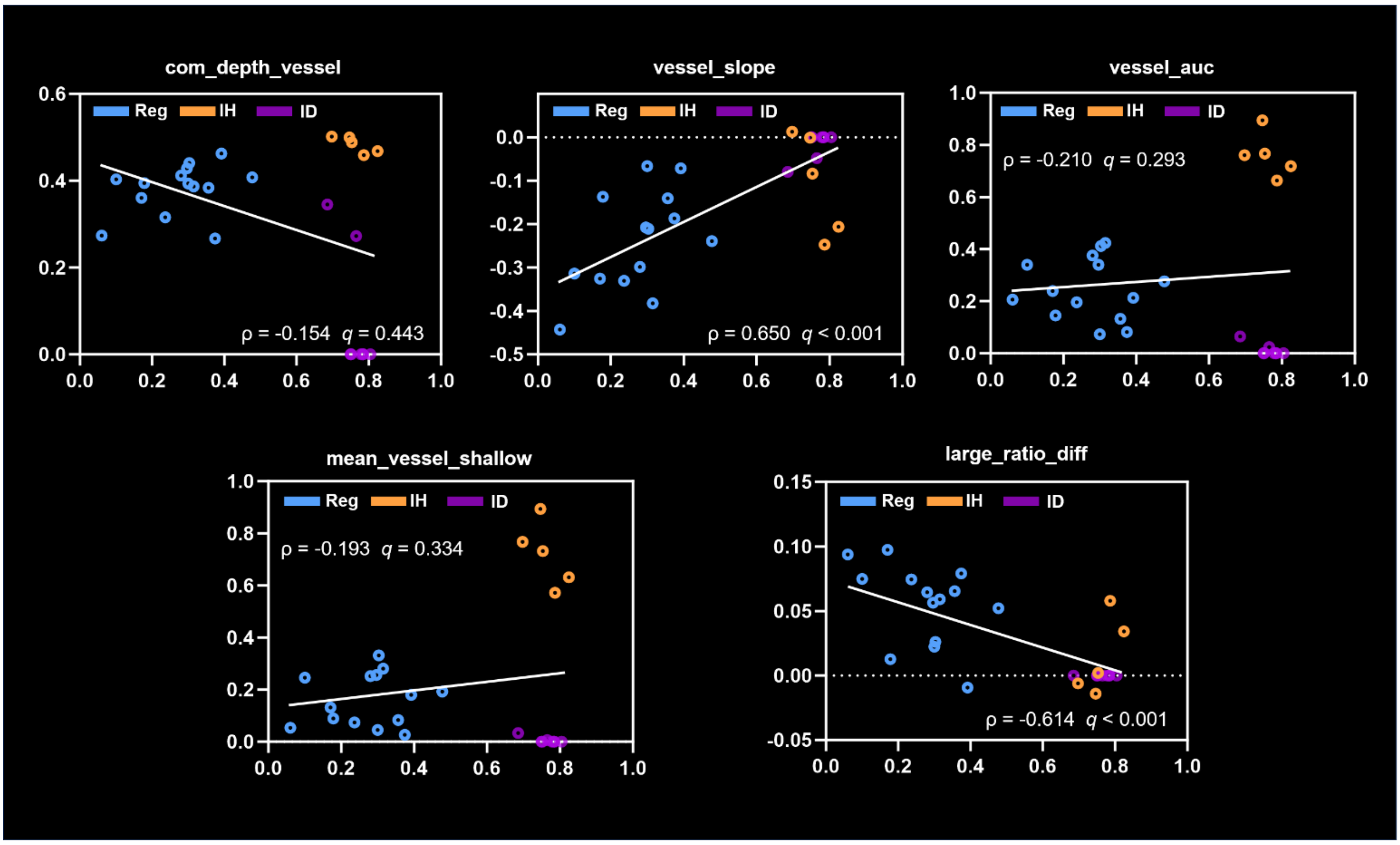


**Figure S2.** Sensitivity analysis of correlations between day-3 D-ULM features and day-14 CAP including all three phenotypes (Reg, IH, and ID). Reg: regenerative phenotype; IH: inflammatory hyperperfusion phenotype; ID: irreversible destructive phenotype.

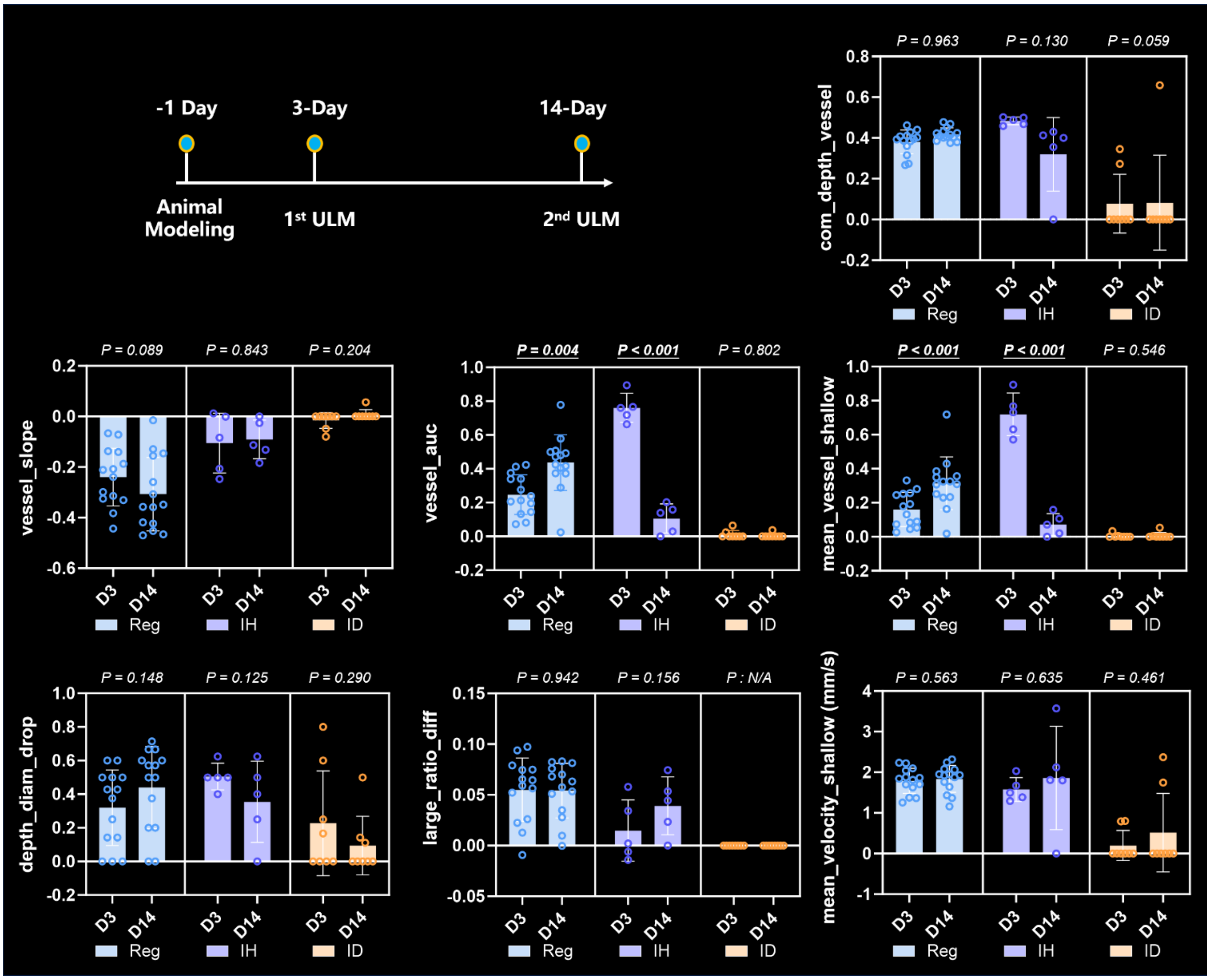


**Figure S3.** Comparison of day-3 and day-14 D-ULM features within each phenotype. Over time, vessel_auc and mean_vessel_shallow were significantly increased in the reg phenotype, whereas they were markedly decreased in the IH phenotype ($P < 0.01$). Sample sizes: n = 14 (Reg), n = 5 (IH), n = 8 (ID). Reg: regenerative phenotype; IH: inflammatory hyperperfusion phenotype; ID: irreversible destructive phenotype.

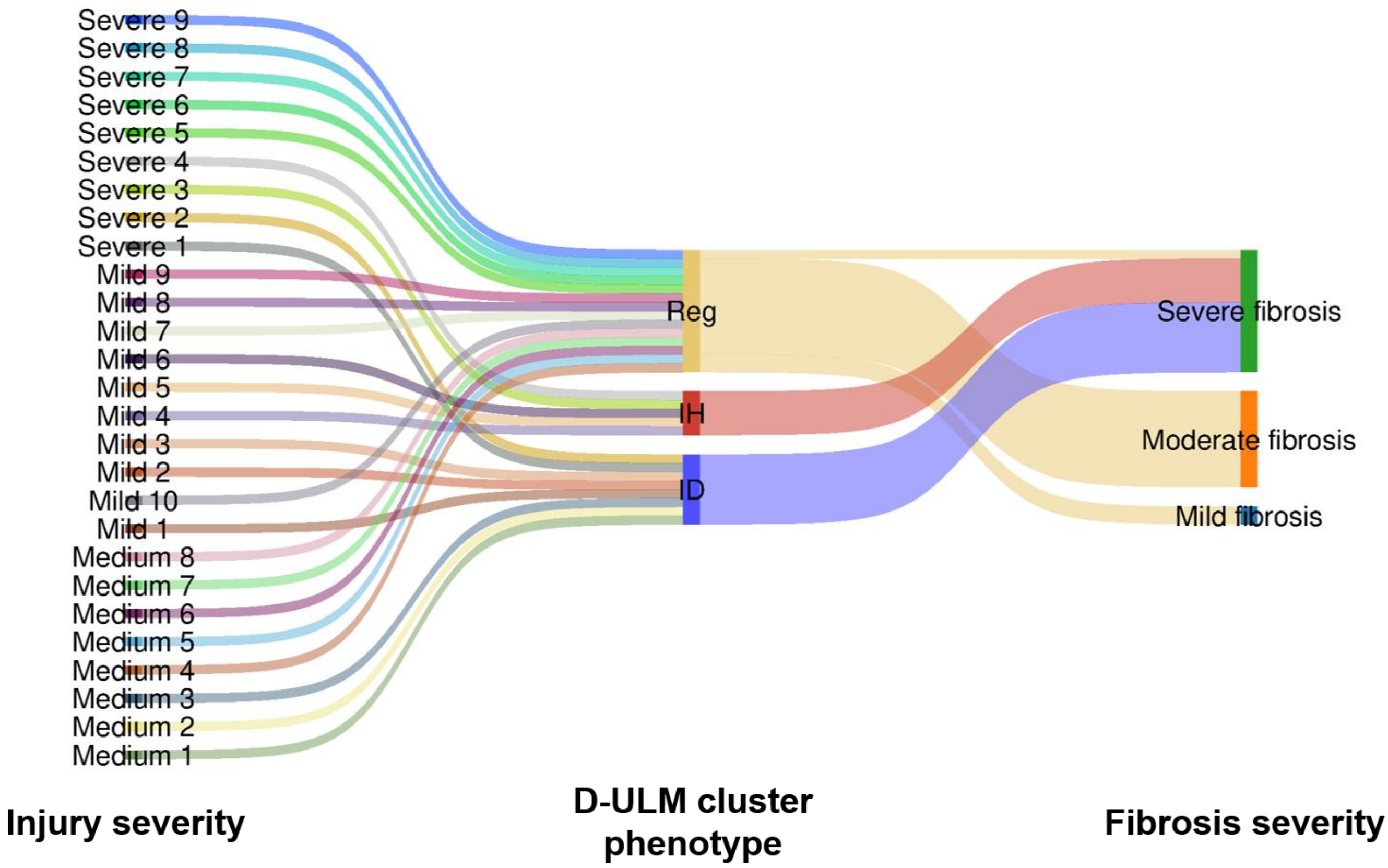


**Figure S4.** Sankey diagram illustrating the distribution of samples across injury severity (mild, moderate, severe), D-ULM cluster phenotype, and fibrotic severity. Injury severity is categorized based on the residence time of 95% ethanol during modeling: mild, 30 s; medium, 50 s; severe, 90 s. According to the predefined criteria, collagen area percentage ranges for mild, moderate, and severe fibrosis are < 10%, 10–50%, and > 50%, respectively. Among the injuries of the same experimental injury severity, D-ULM phenotypes proved useful in distinguishing rats having different fibrotic outcomes, which implies that the D-ULM system reflects biological heterogeneity of microvascular recovery after injury—not fully determined by injury severity. Reg: regenerative phenotype; IH: inflammatory hyperperfusion phenotype; ID: irreversible destructive phenotype.

**Table S1. The unsupervised hierarchical clustering results, priori injury categories and related D-ULM feature values.**

| No. | Expert-based visual category | D-ULM Cluster phenotype | CAP | com_depth_vessel | vessel_slope | vessel_auc | mean_vessel_shallow | depth_diam_drop | large_ratio_diff | mean_velocity_shallow |
|---|---|---|---|---|---|---|---|---|---|---|
| 1 | ID | ID | 0.751 | 0 | 0 | 0 | 0 | 0.167 | 0 | 0 |
| 2 | ID | ID | 0.749 | 0 | 0 | 0 | 0 | 0 | 0 | 0 |
| 3 | ID | ID | 0.784 | 0 | 0 | 0 | 0 | 0 | 0 | 0 |
| 4 | ID | ID | 0.804 | 0 | 0 | 0 | 0 | 0.8 | 0 | 0 |
| 5 | ID | ID | 0.685 | 0.345 | -0.08 | 0.065 | 0.034 | 0.25 | 0 | 0.802 |
| 6 | ID | ID | 0.765 | 0.273 | -0.048 | 0.024 | 0.006 | 0.6 | 0 | 0.794 |
| 7 | ID | ID | 0.784 | 0 | 0 | 0 | 0 | 0 | 0 | 0 |
| 8 | ID | ID | 0.778 | 0 | 0 | 0 | 0 | 0 | 0 | 0 |
| 9 | IH | IH | 0.753 | 0.489 | -0.084 | 0.766 | 0.732 | 0.625 | 0.002 | 2.031 |
| 10 | IH | IH | 0.697 | 0.502 | 0.013 | 0.761 | 0.768 | 0.5 | -0.006 | 1.674 |
| 11 | **IH** | **Reg** | 0.477 | 0.408 | -0.239 | 0.277 | 0.193 | 0.429 | 0.052 | 2.043 |
| 12 | IH | IH | 0.824 | 0.468 | -0.206 | 0.719 | 0.631 | 0.500 | 0.034 | 1.386 |
| 13 | IH | IH | 0.786 | 0.459 | -0.247 | 0.663 | 0.572 | 0.500 | 0.058 | 1.498 |
| 14 | IH | IH | 0.746 | 0.5 | -0.001 | 0.895 | 0.893 | 0.400 | -0.014 | 1.292 |
| 15 | Reg | Reg | 0.303 | 0.441 | -0.211 | 0.412 | 0.331 | 0.600 | 0.026 | 1.707 |
| 16 | Reg | Reg | 0.296 | 0.429 | -0.208 | 0.34 | 0.256 | 0.600 | 0.056 | 1.701 |
| 17 | Reg | Reg | 0.300 | 0.393 | -0.066 | 0.073 | 0.046 | 0 | 0.022 | 1.842 |
| 18 | Reg | Reg | 0.236 | 0.316 | -0.33 | 0.196 | 0.074 | 0.500 | 0.075 | 2.099 |
| 19 | Reg | Reg | 0.170 | 0.360 | -0.325 | 0.240 | 0.132 | 0.375 | 0.097 | 2.221 |
| 20 | Reg | Reg | 0.100 | 0.403 | -0.314 | 0.340 | 0.246 | 0.143 | 0.075 | 1.864 |
| 21 | Reg | Reg | 0.374 | 0.267 | -0.187 | 0.082 | 0.027 | 0.143 | 0.079 | 2.233 |
| 22 | Reg | Reg | 0.178 | 0.395 | -0.137 | 0.145 | 0.089 | 0 | 0.013 | 1.816 |
| 23 | Reg | Reg | 0.392 | 0.462 | -0.072 | 0.213 | 0.180 | 0 | -0.009 | 1.377 |
| 24 | Reg | Reg | 0.315 | 0.387 | -0.382 | 0.424 | 0.281 | 0.250 | 0.059 | 1.880 |
| 25 | Reg | Reg | 0.280 | 0.412 | -0.298 | 0.375 | 0.252 | 0.500 | 0.065 | 1.255 |
| 26 | Reg | Reg | 0.060 | 0.274 | -0.443 | 0.206 | 0.054 | 0.429 | 0.094 | 1.366 |
| 27 | Reg | Reg | 0.356 | 0.384 | -0.141 | 0.132 | 0.084 | 0.500 | 0.065 | 1.626 |

* The expert-based visual categories served as an external validation for the unsupervised clustering. Of note, one rat (No. 11) visually classified as IH was assigned to the Reg phenotype by D-ULM clustering. D-ULM revealed relatively preserved deep vascular signals that may not have been appreciable on visual inspection, highlighting the ability of depth-derived feature analysis to refine phenotype identification. ID: irreversible destructive phenotype; IH: inflammatory hyperperfusion phenotype; Reg: regenerative phenotype; CAP: collagen area percentage.

**Table S2. Spearman correlation analysis between CAP and conventional ROI-based ULM parameters (as a comparison with D-ULM features).**

| Conventional ROI-based ULM parameters | ρ | q value |
|---|---|---|
| Fractal Dimension | 0.354 | 0.137 |
| Vessel Proportion | 0.568 | 0.011 |
| Max Velocity (mm/s) | 0.314 | 0.190 |
| Mean Velocity (mm/s) | -0.147 | 0.547 |
| Tortuosity | 0.509 | 0.026 |
| Mean Diameter (μm) | 0.465 | 0.045 |
| Large Vessel Diameter (μm) | 0.467 | 0.044 |
| Tiny Vessel Diameter (μm) | 0.577 | 0.010 |
| Junction Count | 0.049 | 0.842 |
| Branching Index ($mm^{-2}$) | 0.456 | 0.050 |
